# Estrogens and Progestins Cooperatively Shift Breast Cancer Cell Metabolism

**DOI:** 10.3390/cancers14071776

**Published:** 2022-03-31

**Authors:** Ashley V. Ward, Shawna B. Matthews, Lynsey M. Fettig, Duncan Riley, Jessica Finlay-Schultz, Kiran V. Paul, Matthew Jackman, Peter Kabos, Paul S. MacLean, Carol A. Sartorius

**Affiliations:** 1 Department of Pathology, University of Colorado Anschutz Medical Campus, Aurora, CO 80045, USA; ashley.2.ward@cuanschutz.edu (A.V.W.); shawna.matthews@cuanschutz.edu (S.B.M.); lynsey.fettig@cuanschutz.edu (L.M.F.); duncan.riley@cuanschutz.edu (D.R.); jessica.finlay-schultz@cuanschutz.edu (J.F.-S.); 2 Division of Medical Oncology, Department of Medicine, University of Colorado Anschutz Medical Campus, Aurora, CO 80045, USA; kiran.paul@cuanschutz.edu (K.V.P.); peter.kabos@cuanschutz.edu (P.K.); 3 Division of Endocrinology, Metabolism, and Diabetes, Department of Medicine, University of Colorado Anschutz Medical Campus, Aurora, CO 80045, USA; matthew.jackman@cuanschutz.edu (M.J.); paul.maclean@cuanschutz.edu (P.S.M.)

**Keywords:** estrogens, progestins, estrogen receptor, progesterone receptor, breast cancer, metabolism, glycolysis

## Abstract

**Simple Summary:**

Breast cancers are largely controlled by hormones, expressing abundant receptors for estrogen (ER) and progesterone (PR). Reprogramming of energy metabolism is a common feature of tumors, yet how estrogen and progestins (synthetic progesterone drugs) control breast cancer cell metabolism, particularly in combination, is understudied. Here we evaluated the impact of estrogens and progestins, alone or together, on breast cancer cell metabolism. Our results show that hormones significantly impact metabolism, especially in combination. Estrogens tend to target tumor-promoting genes that alter glucose metabolism while progestins target fat storage. Combined hormone treatment increases both glucose metabolism and fat storage, features advantageous for tumor progression. These results may explain, in part, why estrogen-progestin combinations increase breast cancer incidence in post-menopausal women. Targeting hormone-regulated metabolism is a potential novel therapeutic strategy for ER+PR+ breast cancer.

**Abstract:**

Metabolic reprogramming remains largely understudied in relation to hormones in estrogen receptor (ER) and progesterone receptor (PR) positive breast cancer. In this study, we investigated how estrogens, progestins, or the combination, impact metabolism in three ER and PR positive breast cancer cell lines. We measured metabolites in the treated cells using ultra-performance liquid chromatography coupled with mass spectrometry (UPLC-MS). Top metabolic processes upregulated with each treatment involved glucose metabolism, including Warburg effect/glycolysis, gluconeogenesis, and the pentose phosphate pathway. RNA-sequencing and pathway analysis on two of the cell lines treated with the same hormones, found estrogens target oncogenes, such as MYC and PI3K/AKT/mTOR that control tumor metabolism, while progestins increased genes associated with fatty acid metabolism, and the estrogen/progestin combination additionally increased glycolysis. Phenotypic analysis of cell energy metabolism found that glycolysis was the primary hormonal target, particularly for the progestin and estrogen-progestin combination. Transmission electron microscopy found that, compared to vehicle, estrogens elongated mitochondria, which was reversed by co-treatment with progestins. Progestins promoted lipid storage both alone and in combination with estrogen. These findings highlight the shift in breast cancer cell metabolism to a more glycolytic and lipogenic phenotype in response to combination hormone treatment, which may contribute to a more metabolically adaptive state for cell survival.

## 1. Introduction

The ovarian hormones estradiol and progesterone profoundly impact breast cancer etiology, growth, and treatment. The two hormones frequently act in coordination in target tissues, including the breast. Most breast cancer diagnoses occur in postmenopausal women and three-quarters are estrogen receptor alpha (ER) and usually progesterone receptor (PR) positive [1]. Since circulating levels of hormones are low in these women, tumor growth relies on local adipose tissue production of estrogen [2]. The role of progesterone is more enigmatic given its near-complete dissipation at menopause [3]. However, studies have repeatedly demonstrated that synthetic progestins given in combination with estrogens, versus estrogens alone in menopausal hormone therapy (MHT), increase breast cancer incidence [4]. Endocrine therapies that target ER through competitive ligands, degraders, or blockade of estradiol production remain the cornerstone of breast cancer treatment. More limited studies describe both synthetic progestins and antiprogestins as having some efficacy at attenuating breast cancer growth (reviewed in [5]). Most studies on estrogens and progestins in breast cancer focus on cell proliferation. There are fewer studies on emerging hallmark processes, such as tumor cell metabolism that impact the ability of cells to adapt and survive, particularly in the context of estrogen/progestin combinations.

ER and PR function predominantly as ligand-activated transcriptional factors and are usually co-expressed in the same cells. ER upregulates genes involved in glucose, amino acid, and fatty acid metabolism, while PR increases expression of genes involved in cholesterol/steroid, fatty acid/lipid, and nucleotide/amino acid metabolism [6,7]. Furthermore, both ER and PR upregulate oncogenes, such as MYC, prospectively leading to pleiotropic effects on breast cancer cell metabolism (reviewed in [8,9]). Non-canonical actions of ER and PR may also impact tumor cell metabolism. For example, both receptors have extranuclear signaling activity through rapid activation of kinases, such as PI3K/AKT/mTOR, which could trigger metabolic changes [10,11]. ER and a truncated form of PR (PR-M) have been reported to localize to the mitochondrial matrix and membrane, respectively, in breast cancer cells, and may influence mitochondrial function [12,13,14,15]. One aspect that has been underexplored is the impact of simultaneous ER and PR activation on breast cancer cell bioenergetics and metabolism. This gap is notable as crosstalk between ER and PR has emerged as prototypical in regulating transcription, and measuring proliferation alone may be insufficient to discern distinct metabolic phenotypes that modulate cell survival (reviewed in [16]).

In relation to metabolic pathways, studies have generally found that estrogens increase several energy-generating processes to satisfy the increased energy and biomass needs of growing cells. Estrogens are reported to stimulate oxidative phosphorylation (oxphos) and glycolysis in a cell-line specific manner (reviewed in [6]). Glutamine uptake and consumption have also been found to increase with estrogen treatment in MCF7 breast cancer cells [17]. There are few studies on how progestins affect metabolic pathways in breast cancer. The synthetic progestin medroxyprogesterone acetate (MPA) increases fatty acid synthase in T47D breast cancer cells and increases de novo lipogenesis and accumulation of lipid droplets [18]. There are scarce reports on how estrogen-progestin combinations impact breast cancer cell metabolism specifically, although one study found glucose transporters were increased with the combination in ZR75-1 cells [19]. 

In this study, we performed a comprehensive evaluation of the effect of estrogens, progestins, and the combination on modulation of cell metabolism in ER+PR+ breast cancer cells. We found that each hormone alone had a distinct impact on cell metabolism while the combination consistently increased glycolysis. While estrogens promoted mitochondrial elongation, this was reversed by estrogen-progestin combination. Overall, we conclude that estrogen-progestin combinations have a distinct metabolic phenotype over either hormone alone and may promote more metabolic flexibility. These findings have clinical implications for the use of estrogen-progestin combinations in MHT in postmenopausal women, providing one potential mechanism for increased tumorigenesis, and foster further debate over the use of progestins or antiprogestins in combination with anti-endocrine therapy for breast cancer treatment. 

## 2. Materials and Methods

### 2.1. Cell Lines and Cell Culture Methods

The breast cancer cell line T47D was obtained from the University of Colorado Cancer Center Tissue Culture core and was maintained in minimal Eagle’s medium, 5% fetal bovine serum (FBS), 1× non-essential amino acids, 1 × 10^−9^ M insulin, 0.1 mg/mL sodium pyruvate, and 2 mM L-glutamine. Generation of breast cancer cell lines UCD4 and UCD65 has been previously described [20]. The UCD4 and UCD65 cell lines were maintained in DMEM/F-12 1:1 with 10% FBS, 1 × 10^−9^ M cholera toxin, and 1 × 10^−9^ M insulin. Cell lines were authenticated using short tandem repeat (STR) analysis using the University of Arizona Genetics Core (University of Arizona, Tucson, AZ, USA). All cell lines were routinely tested for mycoplasma contamination using the MycoAlert mycoplasma detection kit (Lonza, Basel, Switzerland). In vitro hormone experiments were performed using phenol red-free media plus charcoal stripped FBS with the same additives described above. Hormone treatment was used as follows: vehicle (0.2% ethanol), 17-β-estradiol (E2), 10^−8^ M (Sigma-Aldrich, St. Louis, MO, USA); R5020, 10^−8^ M (PerkinElmer, Waltham, MA, USA); or progesterone (P4), 10^−7^ M (Sigma-Aldrich), or the combination of E2 plus R5020 (both 10^−8^ M) for 24 h unless otherwise indicated. 

### 2.2. Metabolomics

Metabolites were extracted from T47D, UCD65, and UCD4 cell pellets treated as above with vehicle, E2, R5020, or E2 plus R5020 in quadruplicate using ice-cold lysis/extraction buffer (5:3:2 methanol:acetonitrile:water) at a concentration of 2 × 10^6^ cells per mL. Metabolomics and analyses were performed using the University of Colorado Cancer Center’s Mass Spectrometry Metabolomics Shared Resource, essentially as described [21,22]. Briefly, after sample randomization, 10 μL of extracts were injected into a Thermo Vanquish UHPLC system (San Jose, CA, USA) and resolved on a Kinetex C18 column (150 × 2.1 mm, 1.7 μm, Phenomenex, Torrance, CA, USA) at 450 μL/min through a 5 min gradient from 5 to 95% organic solvent B (mobile phases: A = water, 0.1% formic acid; B = acetonitrile, 0.1% formic acid in positive ion mode or mobile phases: A = 18 mΩ H_2_O, 1 mM ammonium acetate; B = acetonitrile, 1 mM ammonium acetate for negative ion mode). Untargeted data acquisition, quality control, and targeted data analysis were performed as previously described [23]. Metabolomics intensity signals were normalized to cell count per sample. The metabolomics data set supporting the conclusions of this article has been deposited to the MetaboLights, a database for Metabolomics experiments and derived information, with the identifier MTBLS2138 [24]. The complete data set can be accessed the corresponding author.

Normalized data was imported into MetaboAnalyst software [25], where data was log-transformed and autoscaled [26]. Partial least squares discriminant analysis (PLS-DA) was performed on all samples within cell lines for visual inspection of clustering patterns and outlier detection. Heatmaps were constructed using Pearson distance with average linkage and depict non-scaled PLS-DA variable importance in projection (VIP) averaged across replicates (*n* = 4) within treatment groups. 

### 2.3. RNA-Sequencing (RNA-Seq)

Cells were plated in 6 well plates at 5 × 10^5^ (T47D) or 1 × 10^6^ (UCD4) cells/well in phenol red free media and treated with E2, R5020, or the combination as described above for 24 h. RNA was isolated using QIAzol lysis reagent and RNAeasy Mini Kit (Qiagen, Venlo, Netherlands) and treated with RNase-free DNase. Libraries were prepared by the University of Colorado Genomics Shared Resource using Illumina TruSeq Stranded mRNA Library Prep kit and sequenced using paired end 50 cycles 2 × 50 on the NovaSEQ 6000 with >30 million reads per sample obtained. Paired-end 150 nucleotide reads were aligned to the human genome version GRCh38.p13 using STAR. Downstream expression analysis was performed using Cufflinks. The resulting aligned files (BAM) were then used to infer the differentially expressed gene profiles using DESeq2. Pathway analysis was performed using the Molecular Signatures Database hallmark gene set collection. 

### 2.4. Seahorse Metabolic Phenotyping

Metabolic phenotype was determined using the Seahorse Xfe96 Extracellular Flux Analyzer (Agilent, Santa Clara, CA, USA). T47D cells were plated at 1 × 10^4^ cells/well and UCD4/UCD65 cells at 3 × 10^4^ cells/well in the presence of vehicle, E2, R5020, or E2 plus R5020 for 24 h and assessed via the Glycolytic Rate Assay or Mito Stress Test kit (Agilent). Samples were analyzed with 6–8 replicates per treatment. Cell count at time of assay was used for data normalization and obtained using the Cytation 1 Cell Imaging Multi-Mode Reader (BioTek, Santa Clara, CA, USA) with Hoescht 33,342 (Sigma) fluorescent staining.

### 2.5. Transmission Electron Microscopy

Cells were cultured on PermaNox 60-cm dishes (VWR, Radnor, PA, USA) and treated with hormones, as described. For tumor xenografts, 1 × 10^6^ T47D cells were injected into the mammary fat pad of female NOD/SCID/ILIIrg^−/−^ (NSG) mice supplemented with subcutaneous silastic pellets containing E2 or E2 + P4, as previously described [27,28], and collected after 8–10 weeks. All animal experiments were approved by the University of Colorado Institutional Animal Care and Use Committee. Excised tumors were cut into approximately 1 mm^3^ pieces, two tumors per treatment were analyzed. Cultured cells and tumor pieces were fixed with 2% paraformaldehyde and 2.5% glutaraldehyde in 0.1 M phosphate buffer and then post-fixed with reduced osmium (1.5% potassium ferrocyanide + 1% osmium tetroxide) followed by 2% osmium tetroxide. Samples were dehydrated with a graded series of ethanol and embedded in a thin layer of Epon. Following Epon curing, small pieces were cut out and re-embedded in blocks that were sectioned at 65 nm on an ultramicrotome, collected on formvar coated slot grids, and post-stained with 2% osmium tetroxide and lead citrate.

At least 10 fields per treatment were imaged and blinded prior to analysis. Mitochondrial length along the longest axis was measured using Fiji and plotted via histogram, with bin mode indicated on the *X*-axis. Outliers greater than 3 standard deviations outside the mean of the full data set were excluded. Differences in distributions were analyzed using the Kolmogorov–Smirnov test for frequency distributions. 

### 2.6. MitoGFP

T47D cells were labeled overnight with CellLights BacMam 2.0 MitoGFP (Thermo Fisher, Waltham, MA, USA) according to manufacturer’s protocol. Cells were treated for 24 h with vehicle (EtOH), E2 (10^−8^ M) or P4 (10^−8^), or E2 + P4 for 24 h then were fixed in 4% paraformaldehyde, counterstained with DAPI, and mounted on coverslips. Images were collected using confocal laser scanning microscopy (Zeiss LSM 780) with 40× objective. 

### 2.7. Lipid Droplet Staining and Quantitation

T47D or UCD4 cells were plated on glass coverslips at 1.5 × 10^4^ cells/well in phenol-free medium in the presence of vehicle, E2 (10^−8^ M), R5020 (10^−8^ M), or E2 plus R5020 (10^−8^ M each) for 5 days. Coverslips were fixed (10% buffered formalin), stained with Oil Red O in propylene glycol (PEG), counterstained with hematoxylin, mounted on slides, and imaged at 40× magnification (Olympus BX40). Neutral lipid stain was quantified on ImageJ and normalized to cell area (4 fields per condition).

### 2.8. Statistical Analyses

Metabolomics data were analyzed using MetaboAnalyst 5.0 (see Metabolomics section for details). Other statistics were performed using GraphPad Prism 9.3.0 (GraphPad Software, San Diego, CA, USA). Two-tailed Student’s *t*-tests, one-way ANOVA followed by Tukey multiple comparison tests were used to compare groups where noted. Significance at *p* < 0.05 is indicated in figures and legends. 

## 3. Results

### 3.1. Estrogens and Progestins Alter Metabolites in Breast Cancer Cells

We assessed the metabolic impact of hormones in three ER+PR+ breast cancer cell lines: the well characterized T47D line, plus two new cell lines we recently developed, termed UCD4 and UCD65 [20]. T47D cells have wild-type ER and constitutive PR expression. UCD4 cells harbor an activating D538G mutation in ER and have low PR that is modestly increased by estrogens. UCD65 cells have high levels of wild-type ER, and express PR in the absence of estrogens that increases to levels similar to T47D with estrogen treatment [20]. We first measured the impact of estrogens (E2) and the synthetic progestin R5020, alone or in combination, on cell proliferation. Compared to vehicle, E2 increased proliferation in each cell line (modestly in UCD4 likely due to activated ER), while R5020 had variable effects (Supplementary Figure S1). In T47D and UCD4 cells, R5020 did not impact cell growth alone and counteracted E2-stimulated growth. In UCD65 cells, R5020 alone and E2 plus R5020 stimulated growth over vehicle-treated cells. These results underscore the mitogenic effects of estrogens in ER+PR+ breast cancer cells and the well described context specific effects of progestins on cell growth [28]. 

**Figure 1:**
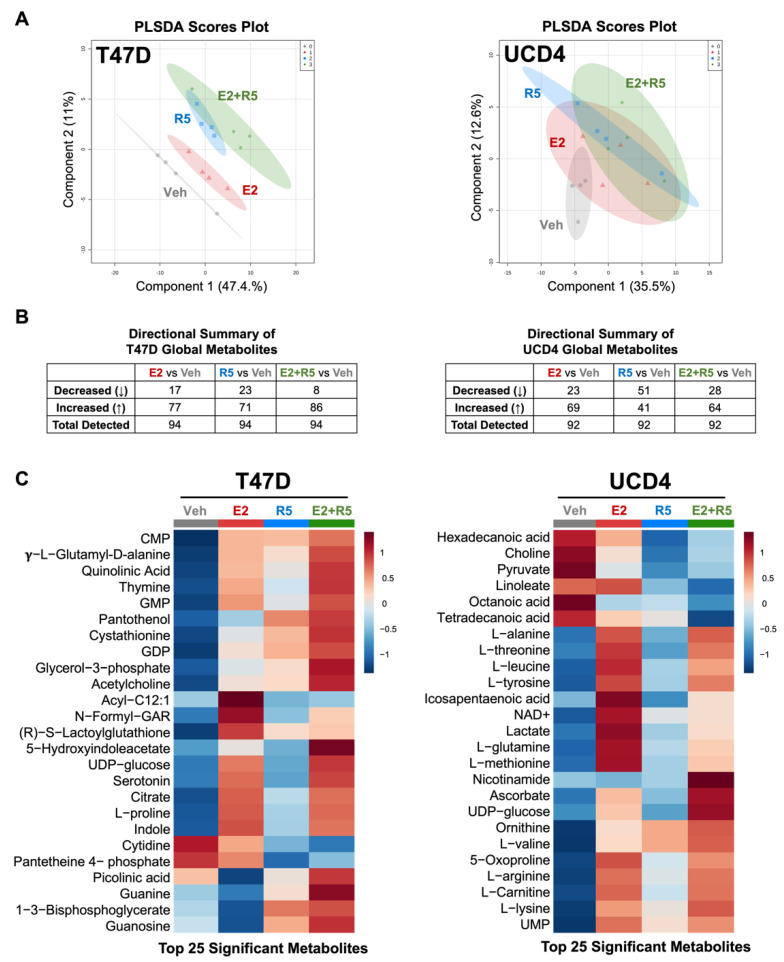
Estrogens and progestins alter global metabolites in T47D and UCD4 breast cancer cells. Untargeted metabolomics was conducted on samples of T47D and UCD4 breast cancer cell lines treated with E2 (10^−8^ M), promegestone (R5, 10^−8^ M), E2 + R5 (10^−8^ M each), or ethanol (Veh) for 24 h (*n* = 4 per treatment condition). Metabolomics data were normalized to cell count, log transformed, and auto-scaled in MetaboAnalyst. (**A**) PLSDA scores plots of T47D or UCD4 samples stratified by hormone treatment. Shaded areas indicate 95% confidence regions; (**B**) directional summary of metabolites for T47D and UCD4 samples with each hormone treatment compared to vehicle. Decreased and increased metabolites were determined by a negative or positive fold change value from vehicle samples, respectively; (**C**) hierarchical clustering heatmaps of the top 25 significant metabolites in T47D and UCD4 samples by ANOVA multiple comparisons. Heatmaps were generated using Euclidean distance measure and Ward clustering method. Samples were averaged by treatment (*n* = 4).

To measure hormone impact on metabolites, we performed global untargeted metabolomics on T47D, UCD4, and UCD65 cells given the same treatments for 24 h. PLS-DA scores plots show that the metabolite signatures of E2, R5020, and E2 plus R5020 differed from vehicle in all three cell lines, but shared some overlap with each other, particularly in the UCD cell lines (Figure 1A, Supplementary Figure S2A). Total metabolites detected for each hormone treatment can be found using MetaboLights Identifier MTBLS2138 [24]. We stratified total metabolites by positive or negative fold change compared to vehicle for each cell line (Figure 1B, Supplementary Figure S2B). E2 increased three times as many total metabolites in each cell line, while R5020 increased total metabolites in two cell lines (T47D, UCD65), and E2 plus R5020 increased relatively more total metabolites in T47D and UCD4 cells. Heatmaps of the top 25 features by variable importance (VIP) scores were generated using MetaboAnalyst (Figure 1C, Supplementary Figure S2C). In T47D and UCD4 cells, E2 and E2 plus R5050 increased most of the top metabolites, while R5020 effects were equivocal (around half of metabolites increased and half were neutral/decreased). Conversely, in UCD65 cells, R5020 produced a robust increase in almost all of the top metabolites. There was minimal overlap in individual hormone-altered top metabolites between cell lines with the exception of UDP-glucose, which was increased by E2 and E2 plus R5020 in T47D and UCD4 cells, and by R5020 in UCD65 cells. Lactate and L-glutamine, and several other L-amino acids increased with hormone treatments in both UCD cell lines. Collectively, these results show that estrogens and progestins alone or in combination significantly alter metabolites in ER+PR+ breast cancer cells, with a trend towards increasing most of the top metabolites. Note that UCD4, with low PR expression, was less affected by R5020 alone. 

### 3.2. Estrogen and Progestins Enrich Metabolites Associated with Glucose Metabolism

**Figure 2:**
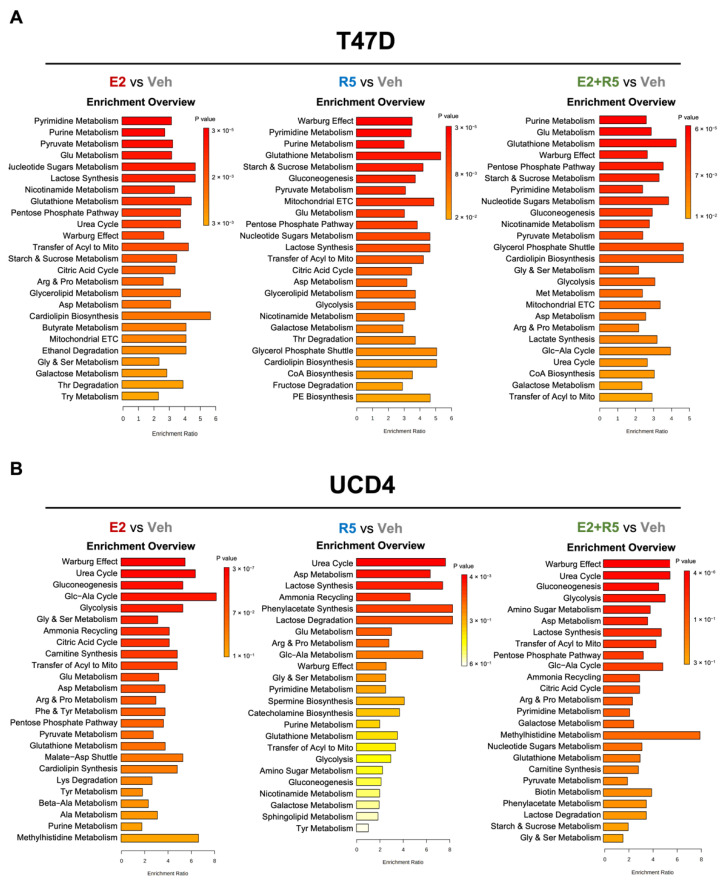
Estrogens and progestins enrich metabolites associated with glucose metabolism and fatty acid metabolism. Metabolite enrichment analysis of T47D (**A**) and UCD4 (**B**) metabolomics samples for each hormone treatment using a positive 1.2−fold change cutoff from vehicle. Bar charts generated using enrichment over representation analysis feature and SMPDB metabolite database on MetaboAnalyst. Enrichment ratio is calculated by the proportion of observed hits to expected hits. Enrichment scores and *p*-values are given in Supplementary Table S1.

To determine metabolic pathways altered by hormones treatments, we utilized the SMPDB metabolite database and enrichment analysis feature in MetaboAnalyst. The top 25 pathways compared to vehicle are indicated for E2, R5020, and E2 plus R5020 for T47D and UCD4 cells (Figure 2A,B) and UCD65 cells (Supplementary Figure S3). Top ranking pathways with E2 treatment shared in all three cell lines include Warburg Effect, Pentose Phosphate Pathway, Citric Acid Cycle, Glutathione metabolism, and metabolism of multiple amino acids. R5020 treatment alone enriched metabolites involved in Warburg Effect, Gluconeogenesis, and Glutathione metabolism in all three cell lines. In addition, R5020-induced fatty acid metabolism in T47D (glycerolipid metabolism) and UCD65 cells (phosphatidylcholine (PC) and phosphatidylethanolamine (PE) biosynthesis). The combination of E2 plus R5020 enriched for most of these processes in addition to glycolysis across the three cell lines. A summary of metabolite pathway analyses is presented in Table S2. Together, these data demonstrate that estrogens and progestins impact multiple metabolic processes in breast cancer cells, with a consistent pattern of elevated glucose metabolic pathways with each hormone alone or together.

### 3.3. Estrogen plus Progestins Target Oncogenes That Alter Metabolism and Fatty Acid Metabolism Genes

**Figure 3:**
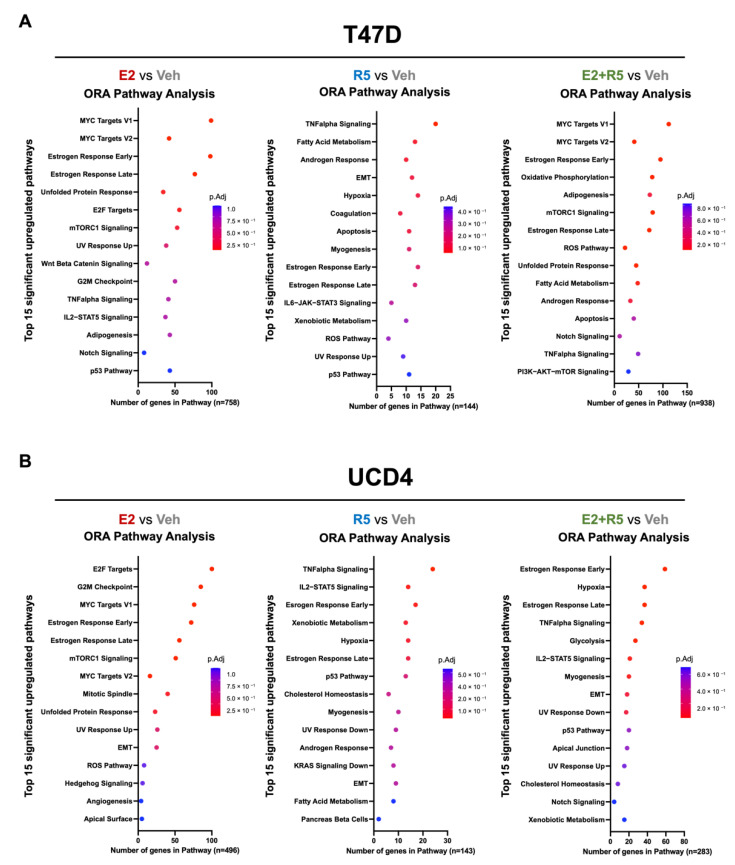
Estrogens and progestins transcriptionally upregulate genes associated with glycolysis, oxidative phosphorylation, and fatty acid metabolism. RNA-seq was conducted on samples of T47D and UCD4 breast cancer cell lines treated with estradiol (E2, 10^−8^ M), promegestone (R5, 10^−8^ M), combination (E2 + R5, 10^−8^ M), or ethanol (Veh) for 24 h (*n* = 3 per treatment condition). （**A**,**B**) Over-representation analysis (ORA) of upregulated pathways in T47D (**A**) and UCD4 (**B**) cells for each hormone treatment compared against vehicle. Analysis conducted in CU Anschutz Genomics Core Shiny App using the Molecular Signatures Database (MSigDB) hallmark gene set collection. Tables of pathways and *p*-values values are given in Supplementary Table S2.

Prior studies have demonstrated the treatment of breast cancer cells with estrogens or progestins alters the expression of genes involved in cell metabolism [8,9]. To interrogate the contribution of gene regulation by estrogen and progestins alone or together on metabolic pathways, we performed RNA-seq on T47D and UCD4 cells following 24 h of hormone treatments. We conducted pathway analysis using the Molecular Signature Database (MSigDB) hallmark gene set collection (Figure 3A,B, Supplementary Table S2). In both T47D and UCD4 cell lines, E2 upregulated MYC and E2F target genes and mTORC1 signaling, each of which regulate the enzymes controlling metabolic nodes, such as glycolysis [29,30]. In both cell lines, R5020 enriched for genes involved in fatty acid metabolism, TNF alpha signaling, and hypoxia. Combination E2 plus R5020 treatment increased transcripts involved in each of the single hormone processes in a cell line specific-manner in addition to adipogenesis and oxphos (T47D cells), and glycolysis (UCD4 cells). In summary, the combination of estrogens plus progestins increases oncogenes that drive metabolic changes in addition to fatty acid metabolism that would support both energy needs and the generation of macromolecules required for growth.

### 3.4. Glycolysis Is Increased by Estrogen plus Progestin Treatment in Breast Cancer Cells

**Figure 4:**
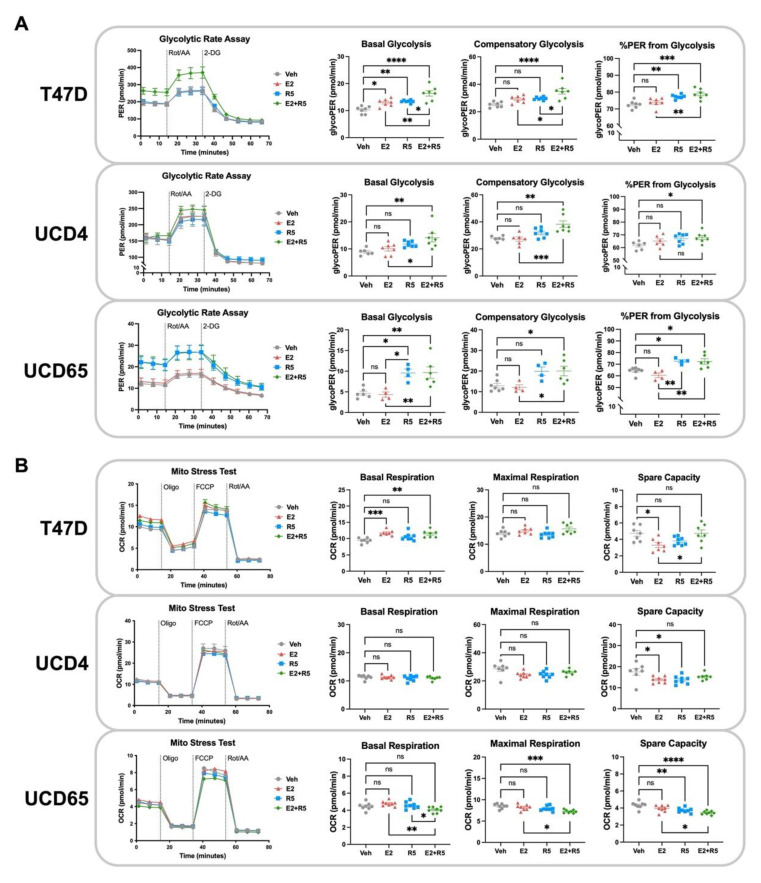
Estrogen plus progestin treatment increases glycolytic activity by Seahorse assay. Glycolytic rate (**A**) and Mito Stress Test (**B**) assays were conducted on T47D, UCD4, and UCD65 cells treated with estradiol (E2, 10^−8^ M), promegestone (R5, 10^−8^ M), combination (E2 + R5, 10^−8^ M each), or ethanol (Veh) for 24 h to assess glycolytic and mitochondrial oxidative activity, respectively (*n* = 5–7 per treatment condition). ns = not significant, * *p* < 0.05, ** *p* < 0.01, *** *p* < 0.001, **** *p* < 0.0001 for the indicated comparisons using one-way ANOVA.

Our metabolomics and RNA-seq data sets implicate alterations in metabolic processes related to energy production are the top targets of hormone treatments in all three cell lines. To further investigate how hormone treatments impact energetic profiles we used the Seahorse Glycolytic Rate Assay and Mito Stress Test to measure glycolysis and oxidative phosphorylation rates, respectively, in T47D, UCD4, and UCD65 cells (Figure 4). In Figure 4A, graphs indicate proton efflux rate (PER) over time (glycolytic rate), basal glycolysis, compensatory glycolysis under the inhibition of complex I and III (Rot/AA) in the electron transport chain, and the percent PER from glycolysis. E2 alone had a limited impact on glycolysis, increasing basal glycolysis only in T47D cells with no impact on compensatory or glycolytic PER. R5020 alone increased basal and compensatory glycolysis, and glycolytic PER in T47D and UCD65 cells (the two cell lines with constitutive PR expression). Interestingly, in all three cell lines, E2 plus R5020 increased each glycolysis measurement compared to vehicle or E2 alone. The Mito Stress Test (Figure 4B) detected increased oxygen consumption only in E2 and E2 plus R5020 treated T47D cells. Hormone treatments did not change or decreased maximal respiration or spare capacity in each cell line. In summary, estrogens alone had little to no impact on glycolysis, progestins increased glycolysis in highly PR+ cell lines, and the combination treatment uniformly augmented glycolytic rate. These results highlight progestins as drivers of glycolysis in combination treatments and the trend towards decreased oxphos suggests a preferential shift towards Warburg-like energy metabolism in combination treated breast cancer cells. 

### 3.5. Progestins Revert Estrogen-Induced Mitochondrial Length in Breast Cancer Cells

**Figure 5:**
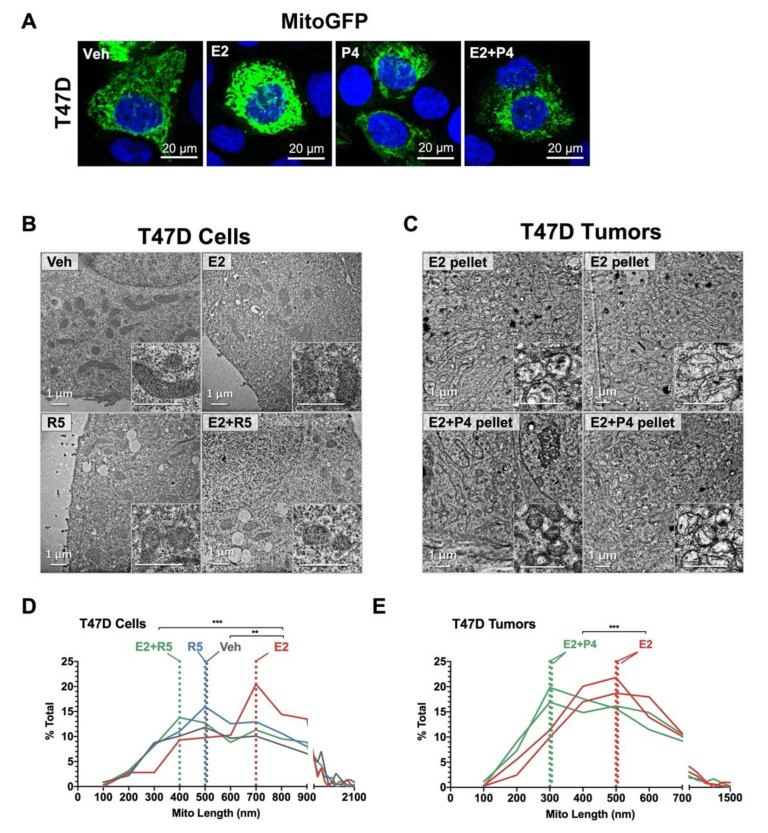
Estrogens elongate while progestins blunt alter mitochondrial length. (**A**) MitoGFP: representative fields from laser scanning confocal images at 40X magnification in T47D cells treated with estradiol (E2, 10^−8^ M), progesterone (P4, 10^−8^ M), combination (E2 + P4, 10^−8^ M each), or ethanol (Veh) for 48 h then labeled overnight with CellLights BacMam 2.0 MitoGFP. Cells were fixed in paraformaldehyde, counterstained with DAPI, and mounted on coverslips. Images were collected using confocal laser scanning microscopy (Zeiss LSM 780) with 40X objective. (**B**) Cultured T47D cells treated with estradiol (E2, 10^−8^ M), promegestone (R5, 10^−8^ M), combination (E2 + R5, 10^−8^ M each), or ethanol (vehicle) for 24 h were fixed, sectioned, and imaged using transmission electron microscopy. A representative image set is presented for each cell line and treatment (top). (**C**) T47D xenograft tumors (2) from mice supplemented with E2 alone or E2 + P4. A representative image set is presented for each tumor and treatment (top). (**D**) Quantitation of mitochondrial length in T47D cells was measured along the longest axis in Fiji in >200 mitochondria across 10–14 fields per treatment. Histograms represent mitochondrial length corresponding to 100-nm bins. T47D cells, mode: E2 + R5 = 400 nm; Veh, R5 = 500 nm, E2 = 700 nm. (**E**) Quantitation of mitochondrial length in T47D tumors (two individual tumors plotted separately) was measured and plotted as in D. T47D tumors, mode: E2 + P4 tumors 1,2 = 300; E2 tumors 1,2 = 500 nm. ** *p* < 0.01, *** *p* < 0.001 for the indicated comparisons using Kolmogorov–Smirnov test for frequency distributions comparing hormone treatments.

Since shifts in cell energy metabolism are often accompanied by altered mitochondria, we examined mitochondrial morphology under different hormone treatments. To grossly visualize mitochondrial networks within cells, T47D cells were labeled with a baculovirus GFP construct (MitoGFP) and then treated for 24 h with hormones. Representative confocal microscopy images are shown in Figure 5A. We observed that E2 treatment, compared to vehicle, generally increased mitochondria network density, while R5020 and E2 plus R5020 did not appear to alter mitochondrial density.

To quantitate mitochondrial morphology, we performed transmission electron microscopy (EM) on T47D cells given different hormone treatments and T47D tumors grown in mice supplemented with E2 vs. E2 plus P4 (Figure 5B). We measured mitochondrial axis length, which can indicate capacity for oxidative respiration (Figure 5C). In T47D cells, E2 treatment increased whereas R5020 did not alter mitochondrial axis length compared to vehicle. In cells treated with E2 plus R5020, mitochondrial length was not different from vehicle, suggesting that R5020 blocks the elongating effect of E2. Mitochondria in T47D tumors supplemented with E2 were more elongated than tumors grown with E2 plus P4 (Figure 5D,E). We observed this same trend in UCD65 cells and UCD4 tumors, with E2 alone increasing mitochondrial length and progestin treatment counteracting E2 affects (Supplementary Figure S4A–D). We also measured mitochondrial turnover in T47D cells using the inducible MitoTimer system (Supplementary Figure S4E–G). Estrogen trended towards increased mitochondrial biogenesis while progestins reversed this effect. These results indicate that while estrogens generally expand mitochondria, progestins blunt expansion of mitochondrial networks in a dominant manner.

### 3.6. Glycerolipid Metabolism Is Increased in Breast Cancer Cells Treated with Progestins and Estrogen/Progestin Combination

**Figure 6:**
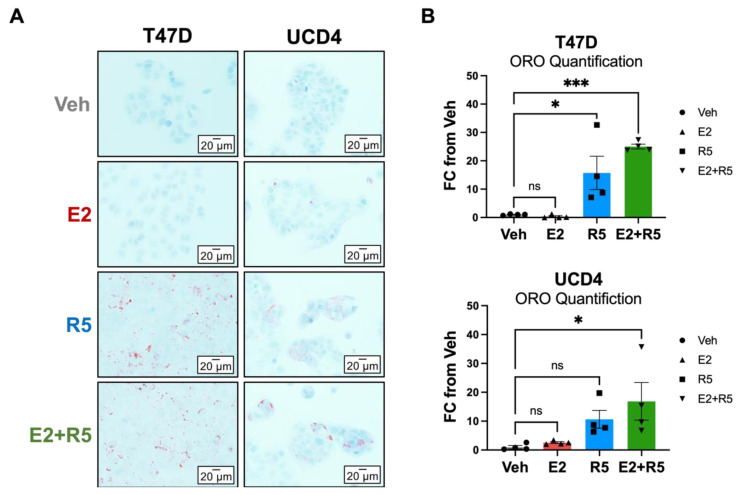
Progestins activate glycerolipid metabolism in breast cancer cell lines. (**A**) Oil Red O (ORO) neutral lipid staining of fixed T47D and UCD4 cells treated with estradiol (E2, 10^−8^ M), promegestone (R5, 10^−8^ M), combination (E2 + R5, 10^−8^ M each), or ethanol (Veh) for 5 days. (**B**) ORO quantification conducted in ImageJ, normalized to cell area, and fold change (FC) compared against vehicle. Four fields were used per treatment condition. * *p* < 0.05, *** *p* < 0.001, ns = not significant.

Progestin treatment was associated with increased fatty acid metabolism by our metabolite and RNA-seq pathway analysis, which has been previously reported in T47D cells [18]. In support of this data, we performed a lipidome analysis of R5020 compared to vehicle treated T47D cells. Glycerolipids (which include storage lipids such as triglycerides and diacylglycerides) increased in abundance in R5020-treated cells (Supplementary Figure S5). To test whether the lipid storage phenotype occurs in other cell models and with estrogen-progestin combination treatment, we stained T47D and UCD4 cells with Oil Red O (ORO), a neutral lipid stain, after 5 days of hormone treatment (Figure 6A). In both cell lines R5020 and E2 plus R5020 robustly increased cytoplasmic lipid droplets compared to vehicle (Figure 6B). These results support previous data that progestins stimulate glycerolipid biosynthesis in ER + PR+ breast cancer cells alone or in tandem with estrogen treatment. 

**Figure 7:**
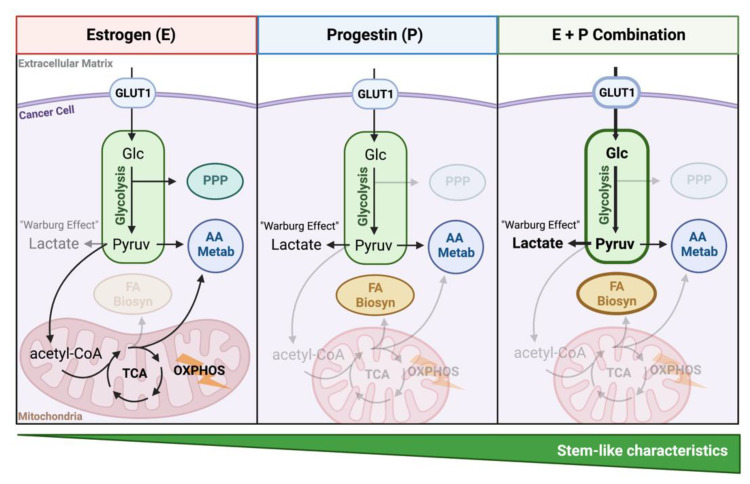
Summary schematic of the metabolic influence of estrogens (E) and progestins (P) on breast cancer cellular metabolism. Estrogens activate oxphos and pentose phosphate pathway (PPP) in a cell line specific manner. Progestins activate glycolysis, lactate production, and fatty acid biosynthesis. The combination further shifts cellular metabolism towards glycolysis and glycerolipid metabolism. Amino acid metabolism, AA Metab; Fatty acid biosynthesis, FA Biosyn; glucose, Glc; Glucose transporter 1, GLUT; oxidative phosphorylation, OXPHOS; pyruvate, Pyruv; tricarbocylic acid cycle, TCA.

The impact of estrogen and progestins on breast cancer cell metabolism is summarized in Figure 7. In the presence of estrogens, ER+PR+ breast cancer cells exhibit a basal level of glycolysis and active oxphos in a cell line specific manner. Amino acid metabolism and pentose phosphate pathways are also active in the presence of estrogen. Progestins preferentially shift cellular metabolism towards glycolysis, lactate production, and condensed mitochondria, all characteristics of the Warburg effect. In addition, progestins activate fatty acid biosynthesis, glycerolipid metabolic pathways, and lipid droplet accumulation. The estrogen plus progestin combination cooperatively enhances glycolysis, fatty acid biosynthesis, and condensed mitochondria, which are reported cancer stem cell (CSC) characteristics. The presence of estrogen in the combination treatment also increases PR expression, enhancing the observed phenotypes.

## 4. Discussion

Metabolic reprogramming is an established hallmark of cancer cells that occurs in conjunction with oncogene activation and transformation. In this study, we investigated the impact of estrogens, progestins, and the combination on global breast cancer cell metabolism in several ER+PR+ cell lines. Hormone treatments significantly altered metabolite profiles with several glucose metabolism pathways including glycolysis, gluconeogenesis, and the pentose phosphate pathway, for example, which were elevated in a hormone and cell-line specific manner. Energetic analysis confirmed that progestins and estrogen-progestin combinations impacted energy metabolism more than estrogen alone, increasing glycolysis with little impact on oxphos. Here we discuss the potential implications of hormone induced shifts in breast cancer cell metabolism with an emphasis on estrogen-progestin combinations, which have not been widely studied.

The seminal discovery that cancer cells activate aerobic glycolysis despite its lower ATP yield (the Warburg effect) has long intrigued scientists [29]. One theory for this phenomenon is that increased expression of glucose transporters in cancer cells leads to ample supplies of glucose for glycolysis and, in addition, provides metabolic intermediates for biosynthetic pathways [30]. A recent multi-omics study conducted on basal, luminal progenitor, and mature luminal cell populations isolated from a normal human breast revealed that basal cells are enriched for glycolysis, while luminal progenitors were enriched for oxphos [31]. Whether these lineage specific metabolic phenotypes persist in breast cancer is unknown. Conventional dogma posits that ER+ breast cancers favor mitochondrial oxphos, while triple negative (TN) breast cancers are highly glycolytic [32,33]. However, estrogens have been described as increasing oxphos and glycolysis in breast cancer cells, although these studies are predominantly limited to the MCF7 cell line [6]. In our metabolite enrichment studies, we found that Warburg effect was significantly altered by estrogens, progestins, and the combination in all three cell lines tested, and glycolysis (or lactate production) was an enriched pathway with progestins and combination treatment. Surprisingly, using functional metabolic analyses, we found that estrogens alone had a limited impact on oxphos or glycolysis, modestly increasing basal glycolysis and respiration only in T47D cells (Figure 4). On the other hand, progestins and estrogens plus progestins significantly increased glycolysis, but not oxphos. Interestingly, progestins blocked estrogen-induced mitochondrial elongation, favoring smaller, rounder mitochondria in cell lines and tumors (Figure 5), which is consistent with lower functional capacity and glycolysis [34]. These results highlight that glycolysis is specifically targeted by progestins alone or with estrogens. Estrogens may have a more significant impact on other glucose metabolic processes not directly measured in our studies.

The impact of progestins on breast cancer cell metabolism alone or with estrogens is particularly interesting, as this has been rarely studied. Progesterone is the key hormone necessary for mammary stem cell fluxes during the estrous cycle and in pregnancy where E2 and P4 are both present (reviewed in [35,36]). In breast cancer, progestins increase mammosphere and tumor initiation, properties of CSCs [37]. While CSCs show considerable metabolic plasticity, in many cases they preferentially utilize glycolysis, prospectively to avoid damaging effects of reactive oxygen species generation during oxphos [38]. Notably, glutathione metabolism, which is elevated in breast CSCs as an antioxidant defense [39], was increased by metabolite analysis with all hormone treatments including estrogen plus progestins. By metabolite analysis and RNA-seq, progestins alone or with estrogens altered fatty acid metabolism. The progestin MPA was previously found to increase expression of stearoyl-CoA desaturase (SCD-1), a lipogenic enzyme that synthesizes monounsaturated fatty acids, and lipid droplets in T47D cells [18]. We confirmed that progestins, alone or with estrogens, increase cytosolic lipid droplet accumulation in T47D and UCD4 breast cancer cells (Figure 6), a feature recently associated with CSCs [40]. Together, the combination of estrogen plus progestin imparts a unique metabolic phenotype that has been associated with cancer cell metabolic resiliency. 

We utilized three breast cancer cell lines that, while they share ER+PR+ status, are heterogeneous in their oncogene mutations and growth response to hormones. For example, UCD4 harbors a homozygous somatic activating mutation in the ligand binding domain of ER (D538G) [20]. Therefore, UCD4 is relatively less estrogen responsive than the other cell lines, exhibiting baseline growth without E2 supplementation in vitro and in vivo (Supplementary Figure S1) [28]. Interestingly, exogenously expressed Y537S and D538G ER mutants in MCF7 cells have been associated with increased oxphos with or without glycolysis [41,42]. In UCD4 cells, hormones had no effect on oxphos, but increased glycolysis with estrogen plus progestins (Figure 4). We speculate this is due to estrogen induction of PR that facilitates progestin-induced glycolysis. UCD65 cells were unique in that both estrogens and progestins stimulated growth and progestins had a more profound impact on metabolites (Figure S2). We have observed that progestins attenuate estrogen driven growth of UCD65 tumors in vivo [28], and speculate some of these effects could be through the tumor microenvironment. ER and PR target several oncogenes that are known to impact tumor cell metabolism including MYC and PI3K/AKT/mTOR that increase glucose transport, glycolysis, and lipid synthesis. By RNA-seq, progestin treated cells were enriched for hypoxia, which is indicative of glucose to lactate conversion during glycolysis. Progestins increased TNF alpha signaling, a cytokine involved in both lipid metabolism and breast cancer progression [43]. Collectively, our studies implicate that ER and PR signaling produces an extremely flexible metabolic phenotype. 

There are clear implications for estrogen plus progestins altering breast cancer cell metabolism. Incidence rates of ER+PR+, as opposed to TN breast cancer, continue to climb with age and constitute >80% of diagnoses in women >50 [44]. At menopause, the major source of estrogen production transitions to adipose tissue. Obesity is a specific risk factor for ER+PR+ breast cancer in postmenopausal women, prospectively due to increased adipose estrogen production [45]. It is important to note that estrone (E1) is the dominant estrogen produced post menopause and has been associated with increased breast cancer incidence in postmenopausal obesity [46]. Thus, future studies on the impact of E1 vs. E2 on breast cancer cell metabolism could shed light on the increased prevalence of ER+ breast cancer in post vs. premenopausal women. PR was shown to be central to oncogenesis in a rat model of obesity and mammary tumorigenesis [47]. The contribution of progestins to breast cancer incidence was revealed by large trials on MHT. Surprisingly, the use of estrogens alone was not associated with significantly increased breast cancer risk, but rather estrogen plus progestin therapy (EPT) significantly increased the incidence of ER+PR+ breast cancer [48]. These observations have been repeatedly observed with multiple synthetic progestins, while the role of the natural hormone progesterone remains under debate [4,49]. Moreover, mechanisms for EPT-induced breast cancer are speculated to involve awakening of dormant tumor cells and/or expansion of CSCs [50,51]. Our studies support that EPT targets glycolysis and a lipid biogenesis phenotype in breast cancer cells, which are both reportedly enhanced in breast CSCs. We, therefore, speculate that the EPT combination may cause metabolic flexibility in early tumor cells that could trigger their expansion. EPT exposure in existing breast cancers could also be detrimental as a meta-analysis found that tumors expressing high levels of glycolytic proteins corresponded to shorter overall survival of breast cancer patients [52]. Anti-progestins are being explored for treatment of metastatic ER+ breast cancer [53], although their role in modulation of breast cancer metabolism is unknown.

## 5. Conclusions

Estrogens and progestins are commonly used in women’s medicines, often in combination, and are involved in breast cancer tumorigenesis and progression. Our studies support that estrogens plus progestins target tumor cell metabolism in ER+PR+ breast cancer cells by simultaneously increasing both glycolysis and lipid storage. Surprisingly, progestins appeared to dominantly drive the metabolic phenotype, with estrogens necessary to boost PR in some cell lines. Several other processes, including glutathione antioxidant defense, heightened with estrogen-progestin treatment. Interestingly, each of these processes is reportedly heightened in CSCs. We conclude that EPT may impart metabolic advantages to cancer cells that are not evident by measuring proliferation alone. These observations highlight potential mechanisms of EPT-induced tumorigenesis at menopause and suggest that targeting hormone-driven metabolic phenotypes, particularly lipid metabolism, which has seen some promising recent advances, could be a novel therapeutic vulnerability.

## Data Availability

The metabolomics data set has been deposited to the MetaboLights, a database for Metabolomics experiments and derived information, with the identifier MTBLS2138, and the complete data set can be accessed the corresponding author. The RNA-seq data set can be accessed using reference number GSE196880 at the Gene Expression Omnibus (GEO) database repository, https://www.ncbi.nlm.nih.gov/geo/query/acc.cgi?acc=GSE196880 (accessible on 31 March 2022).

## Supplementary Materials

The following supporting information can be downloaded at: https://www.mdpi.com/article/10.3390/cancers14071776/s1, Figure S1: Proliferation of breast cancer cell lines with hormone treatments; Figure S2: Steroid hormones increase global metabolites in UCD65 breast cancer cell lines; Figure S3: Metabolite enrichment analysis of UCD65 cells; Figure S4: Steroid hormones alter mitochondrial morphology and length in UCD65 cells and UCD4 tumors; Figure S5: Progestin treatment increases total glycerolipids compared to vehicle in T47D cells; Table S1: T47D and UCD4 Metabolite Enrichment Tables; Table S2: T47D and UCD4 RNA-seq ORA Tables.

## References

[1] Jatoi I., Chen B.E., Anderson W.F., Rosenberg P.S. (2007). Breast cancer mortality trends in the United States according to estrogen receptor status and age at diagnosis. J. Clin. Oncol..

[2] Siiteri P.K. (1987). Adipose tissue as a source of hormones. Am. J. Clin. Nutr..

[3] Santoro N., Randolph J.F. (2011). Reproductive hormones and the menopause transition. Obstet. Gynecol. Clin. N. Am..

[4] Collaborative Group on Hormonal Factors in Breast Cancer (2019). Type and timing of menopausal hormone therapy and breast cancer risk: Individual participant meta-analysis of the worldwide epidemiological evidence. Lancet.

[5] Carroll J.S., Hickey T.E., Tarulli G.A., Williams M., Tilley W.D. (2017). Deciphering the divergent roles of progestogens in breast cancer. Nat. Rev. Cancer.

[6] Boonyaratanakornkit V., Pateetin P. (2015). The role of ovarian sex steroids in metabolic homeostasis, obesity, and postmenopausal breast cancer: Molecular mechanisms and therapeutic implications. BioMed Res. Int..

[7] Hussein S., Khanna P., Yunus N., Gatza M.L. (2021). Nuclear Receptor-Mediated Metabolic Reprogramming and the Impact on HR+ Breast Cancer. Cancers.

[8] Frasor J., Danes J.M., Komm B., Chang K.C., Lyttle C.R., Katzenellenbogen B.S. (2003). Profiling of estrogen up- and down-regulated gene expression in human breast cancer cells: Insights into gene networks and pathways underlying estrogenic control of proliferation and cell phenotype. Endocrinology.

[9] Richer J.K., Jacobsen B.M., Manning N.G., Abel M.G., Wolf D.M., Horwitz K.B. (2002). Differential gene regulation by the two progesterone receptor isoforms in human breast cancer cells. J. Biol. Chem..

[10] Boonyaratanakornkit V., McGowan E., Sherman L., Mancini M.A., Cheskis B.J., Edwards D.P. (2007). The role of extranuclear signaling actions of progesterone receptor in mediating progesterone regulation of gene expression and the cell cycle. Mol. Endocrinol..

[11] Diep C.H., Daniel A.R., Mauro L.J., Knutson T.P., Lange C.A. (2015). Progesterone action in breast, uterine, and ovarian cancers. J. Mol. Endocrinol..

[12] Chen J.Q., Delannoy M., Cooke C., Yager J.D. (2004). Mitochondrial localization of ERalpha and ERbeta in human MCF7 cells. Am. J. Physiol. Endocrinol. Metab..

[13] Pedram A., Razandi M., Wallace D.C., Levin E.R. (2006). Functional estrogen receptors in the mitochondria of breast cancer cells. Mol. Biol. Cell.

[14] Behera M.A., Dai Q., Garde R., Saner C., Jungheim E., Price T.M. (2009). Progesterone stimulates mitochondrial activity with subsequent inhibition of apoptosis in MCF-10A benign breast epithelial cells. Am. J. Physiol. Endocrinol. Metab..

[15] Dai Q., Shah A.A., Garde R.V., Yonish B.A., Zhang L., Medvitz N.A., Miller S.E., Hansen E.L., Dunn C.N., Price T.M. (2013). A truncated progesterone receptor (PR-M) localizes to the mitochondrion and controls cellular respiration. Mol. Endocrinol..

[16] Tidwell T.R., Soreide K., Hagland H.R. (2017). Aging, Metabolism, and Cancer Development: From Peto’s Paradox to the Warburg Effect. Aging Dis..

[17] Forbes N.S., Meadows A.L., Clark D.S., Blanch H.W. (2006). Estradiol stimulates the biosynthetic pathways of breast cancer cells: Detection by metabolic flux analysis. Metab. Eng..

[18] Schlaepfer I.R., Hitz C.A., Gijon M.A., Bergman B.C., Eckel R.H., Jacobsen B.M. (2012). Progestin modulates the lipid profile and sensitivity of breast cancer cells to docetaxel. Mol. Cell. Endocrinol..

[19] Medina R.A., Meneses A.M., Vera J.C., Guzman C., Nualart F., Astuya A., Garcia M.A., Kato S., Carvajal A., Pinto M. (2003). Estrogen and progesterone up-regulate glucose transporter expression in ZR-75-1 human breast cancer cells. Endocrinology.

[20] Finlay-Schultz J., Jacobsen B.M., Riley D., Paul K.V., Turner S., Ferreira-Gonzalez A., Harrell J.C., Kabos P., Sartorius C.A. (2020). New generation breast cancer cell lines developed from patient-derived xenografts. Breast Cancer Res..

[21] D’Alessandro A., Moore H.B., Moore E.E., Wither M., Nemkov T., Gonzalez E., Slaughter A., Fragoso M., Hansen K.C., Silliman C.C. (2015). Early hemorrhage triggers metabolic responses that build up during prolonged shock. Am. J. Physiol. Regul. Integr. Comp. Physiol..

[22] D’Alessandro A., Nemkov T., Kelher M., West F.B., Schwindt R.K., Banerjee A., Moore E.E., Silliman C.C., Hansen K.C. (2015). Routine storage of red blood cell (RBC) units in additive solution-3: A comprehensive investigation of the RBC metabolome. Transfusion.

[23] Gehrke S., Rice S., Stefanoni D., Wilkerson R.B., Nemkov T., Reisz J.A., Hansen K.C., Lucas A., Cabrales P., Drew K. (2019). Red Blood Cell Metabolic Responses to Torpor and Arousal in the Hibernator Arctic Ground Squirrel. J. Proteome Res..

[24] Haug K., Cochrane K., Nainala V.C., Williams M., Chang J., Jayaseelan K.V., O’Donovan C. (2020). MetaboLights: A resource evolving in response to the needs of its scientific community. Nucleic Acids Res..

[25] Chong J., Wishart D.S., Xia J. (2019). Using MetaboAnalyst 4.0 for Comprehensive and Integrative Metabolomics Data Analysis. Curr. Protoc. Bioinform..

[26] Xia J., Wishart D.S. (2011). Metabolomic data processing, analysis, and interpretation using MetaboAnalyst. Curr. Protoc. Bioinform..

[27] Fettig L.M., McGinn O., Finlay-Schultz J., LaBarbera D.V., Nordeen S.K., Sartorius C.A. (2017). Cross talk between progesterone receptors and retinoic acid receptors in regulation of cytokeratin 5-positive breast cancer cells. Oncogene.

[28] Finlay-Schultz J., Gillen A.E., Brechbuhl H.M., Ivie J.J., Matthews S.B., Jacobsen B.M., Bentley D.L., Kabos P., Sartorius C.A. (2017). Breast Cancer Suppression by Progesterone Receptors Is Mediated by Their Modulation of Estrogen Receptors and RNA Polymerase III. Cancer Res..

[29] Warburg O. (1956). On the origin of cancer cells. Science.

[30] Ge T., Yang J., Zhou S., Wang Y., Li Y., Tong X. (2020). The Role of the Pentose Phosphate Pathway in Diabetes and Cancer. Front. Endocrinol..

[31] Mahendralingam M.J., Kim H., McCloskey C.W., Aliar K., Casey A.E., Tharmapalan P., Pellacani D., Ignatchenko V., Garcia-Valero M., Palomero L. (2021). Mammary epithelial cells have lineage-rooted metabolic identities. Nat. Metab..

[32] Klinge C.M. (2008). Estrogenic control of mitochondrial function and biogenesis. J. Cell. Biochem..

[33] Avagliano A., Ruocco M.R., Aliotta F., Belviso I., Accurso A., Masone S., Montagnani S., Arcucci A. (2019). Mitochondrial Flexibility of Breast Cancers: A Growth Advantage and a Therapeutic Opportunity. Cells.

[34] Liesa M., Shirihai O.S. (2013). Mitochondrial dynamics in the regulation of nutrient utilization and energy expenditure. Cell Metab..

[35] Axlund S.D., Sartorius C.A. (2012). Progesterone regulation of stem and progenitor cells in normal and malignant breast. Mol. Cell. Endocrinol..

[36] Simoes B.M., Alferez D.G., Howell S.J., Clarke R.B. (2015). The role of steroid hormones in breast cancer stem cells. Endocr.-Relat. Cancer.

[37] Horwitz K.B., Sartorius C.A. (2020). 90 YEARS OF PROGESTERONE: Progesterone and progesterone receptors in breast cancer: Past, present, future. J. Mol. Endocrinol..

[38] Batlle E., Clevers H. (2017). Cancer stem cells revisited. Nat. Med..

[39] Luo M., Shang L., Brooks M.D., Jiagge E., Zhu Y., Buschhaus J.M., Conley S., Fath M.A., Davis A., Gheordunescu E. (2018). Targeting Breast Cancer Stem Cell State Equilibrium through Modulation of Redox Signaling. Cell Metab..

[40] Mancini R., Noto A., Pisanu M.E., De Vitis C., Maugeri-Sacca M., Ciliberto G. (2018). Metabolic features of cancer stem cells: The emerging role of lipid metabolism. Oncogene.

[41] Fiorillo M., Sanchez-Alvarez R., Sotgia F., Lisanti M.P. (2018). The ER-alpha mutation Y537S confers Tamoxifen-resistance via enhanced mitochondrial metabolism, glycolysis and Rho-GDI/PTEN signaling: Implicating TIGAR in somatic resistance to endocrine therapy. Aging.

[42] Zinger L., Merenbakh-Lamin K., Klein A., Elazar A., Journo S., Boldes T., Pasmanik-Chor M., Spitzer A., Rubinek T., Wolf I. (2019). Ligand-binding Domain-activating Mutations of ESR1 Rewire Cellular Metabolism of Breast Cancer Cells. Clin. Cancer Res..

[43] Cruceriu D., Baldasici O., Balacescu O., Berindan-Neagoe I. (2020). The dual role of tumor necrosis factor-alpha (TNF-alpha) in breast cancer: Molecular insights and therapeutic approaches. Cell. Oncol..

[44] Benz C.C. (2008). Impact of aging on the biology of breast cancer. Crit. Rev. Oncol. Hematol..

[45] Sahin S., Erdem G.U., Karatas F., Aytekin A., Sever A.R., Ozisik Y., Altundag K. (2017). The association between body mass index and immunohistochemical subtypes in breast cancer. Breast.

[46] Qureshi R., Picon-Ruiz M., Aurrekoetxea-Rodriguez I., Nunes de Paiva V., D’Amico M., Yoon H., Radhakrishnan R., Morata-Tarifa C., Ince T., Lippman M.E. (2020). The Major Pre- and Postmenopausal Estrogens Play Opposing Roles in Obesity-Driven Mammary Inflammation and Breast Cancer Development. Cell Metab..

[47] Giles E.D., Wellberg E.A., Astling D.P., Anderson S.M., Thor A.D., Jindal S., Tan A.C., Schedin P.S., Maclean P.S. (2012). Obesity and overfeeding affecting both tumor and systemic metabolism activates the progesterone receptor to contribute to postmenopausal breast cancer. Cancer Res..

[48] Chlebowski R.T., Anderson G.L., Gass M., Lane D.S., Aragaki A.K., Kuller L.H., Manson J.E., Stefanick M.L., Ockene J., Sarto G.E. (2010). Estrogen plus progestin and breast cancer incidence and mortality in postmenopausal women. JAMA.

[49] Chlebowski R.T., Anderson G.L., Aragaki A.K., Manson J.E., Stefanick M.L., Pan K., Barrington W., Kuller L.H., Simon M.S., Lane D. (2020). Association of Menopausal Hormone Therapy With Breast Cancer Incidence and Mortality During Long-term Follow-up of the Women’s Health Initiative Randomized Clinical Trials. JAMA.

[50] Santen R.J. (2003). Risk of breast cancer with progestins: Critical assessment of current data. Steroids.

[51] Horwitz K.B., Sartorius C.A. (2008). Progestins in hormone replacement therapies reactivate cancer stem cells in women with preexisting breast cancers: A hypothesis. J. Clin. Endocrinol. Metab..

[52] Yu M., Chen S., Hong W., Gu Y., Huang B., Lin Y., Zhou Y., Jin H., Deng Y., Tu L. (2019). Prognostic role of glycolysis for cancer outcome: Evidence from 86 studies. J. Cancer Res. Clin. Oncol..

[53] Kamaraju S., Fowler A.M., Weil E., Wisinski K.B., Truong T.H., Lehr M., Chaudhary L.N., Cheng Y.C., Chitambar C.R., Rui H. (2021). Leveraging Antiprogestins in the Treatment of Metastatic Breast Cancer. Endocrinology.

