# Supplementary material for: Estrogens and Progestins Cooperatively Shift Breast Cancer Cell Metabolism
Source: Cancers (Basel). 2022 Mar 31;14(7):1776. doi: 10.3390/cancers14071776 (PMC8996926; doi:10.3390/cancers14071776)
Supplement: Supplementary file 1 [file cancers-14-01776-s001.zip › cancers-1635104-supplementary.pdf]

cancers

MDPI

Supplementary Materials

Estrogens and progestins cooperatively shift breast cancer cell metabolism

Ashley V. Ward, Shawna B. Matthews, Lynsey M. Fettig, Duncan Riley, Jessica Finlay-Schultz, Kiran V. Paul, Matthew Jackman, Peter Kabos, Paul S. MacLean and Carol A. Sartorius

Supplementary Figure S1

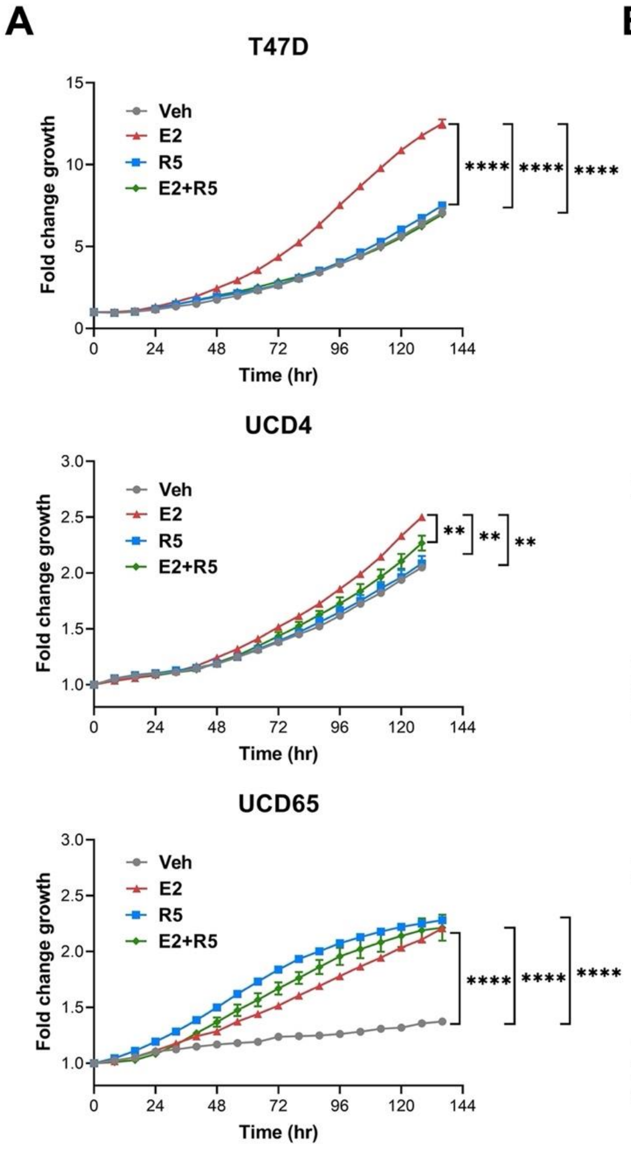

Figure S1. Proliferation of breast cancer cell lines with hormone treatments. A) T47D, UCD4, and UCD65 cells labeled with nuclear GFP were plated in 96 well plated and treated with Vehicle (Veh), E2 (10 nM), R5020 (R5, 10 nM), or the combination (10 nm each) and monitored for green object counts (cell number) using the Incucyte Live Cell Imaging system (Sartorius). **B**) Cell proliferation plotted at 24 h timepoint. N=6−12 per cell line/hormone condition, mean fold change over vehicle ± SEM are indicated. Two-way ANOVA/Tukey multiple comparisons are indicated where $P<0.05$. * $p < 0.05$, ** $p < 0.01$, **** $p < 0.0001$ for the indicated comparisons using one-way ANOVA.

Supplementary Figure S2

A

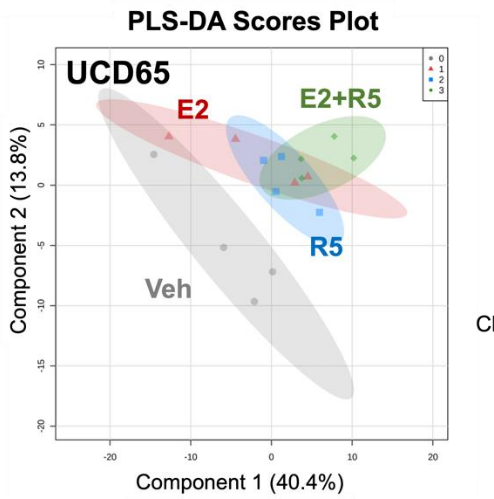

C

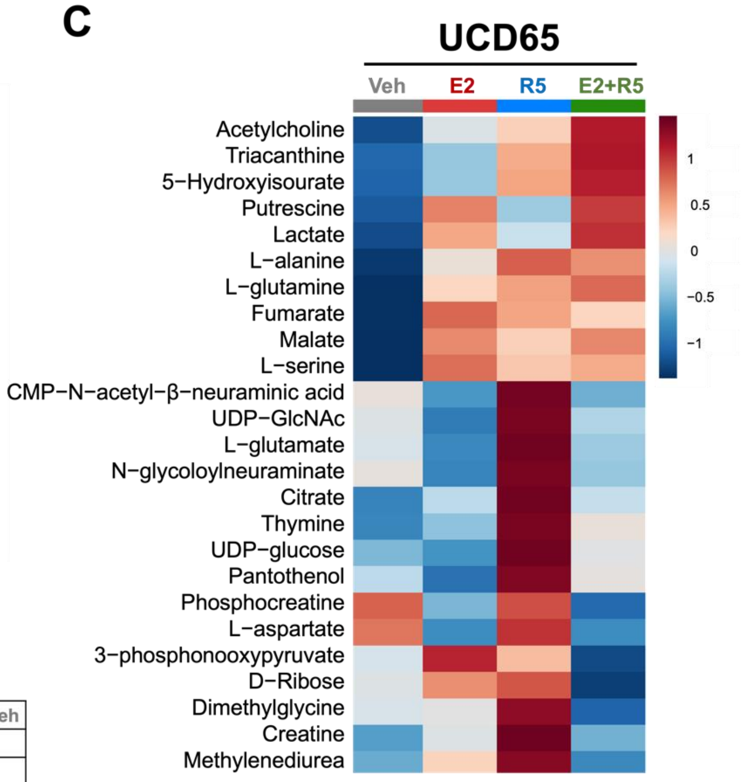

B

Directional Summary of UCD4 Global Metabolites

	E2 vs Veh	R5 vs Veh	E2+R5 vs Veh
**Decreased (↓)**	37	20	70
**Increased (↑)**	104	121	71
**Total Detected**	141	141	141

Figure S2. Steroid hormones increase global metabolites in UCD65 breast cancer cell lines. Untargeted metabolomics were conducted on UCD65 cells treated with estradiol (E2, 10nM), promegesterone (R5, 10nM), combination (E2+R5, 10nM/10nM), or ethanol (Veh) for 24 h. Data was normalized to total protein per sample, log transformed, and auto-scaled in Metaboanalyst. **A**) Partial Least Squares Discriminant Analysis (PLS-DA) scores plot of total metabolites detected in UCD65 cells with hormone treatment. Shaded regions represent 95% confidence region. **B**) Directional summary of metabolites for UCD65 cell samples within each hormone treatment. Decreased and increased metabolites were determined by a negative or positive fold change value from vehicle samples, respectively. **C**) Heatmap of top 25 metabolites of hormone treated UCD65 cells by ANOVA multiple comparisons. Samples were averaged by treatment (n=4) and Euclidean distance measure and Ward clustering methods were used to generate heatmaps.

Supplementary Figure S3.

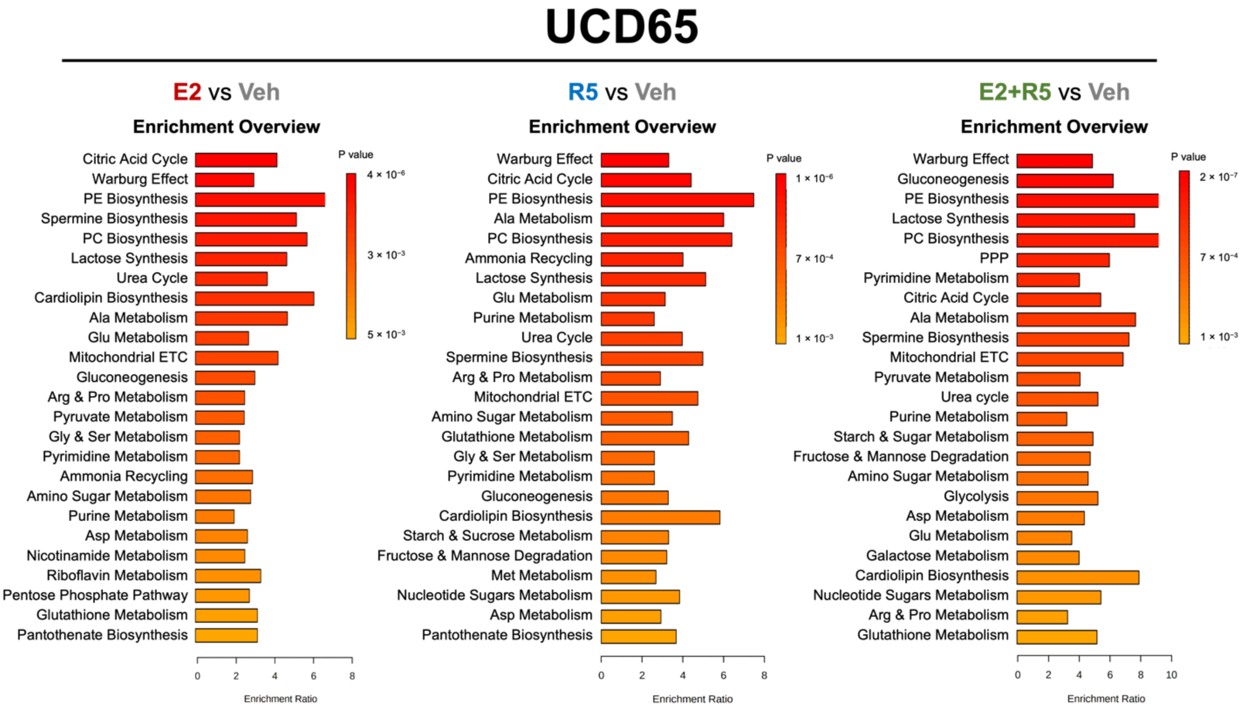

Figure S3. Metabolite enrichment analysis of UCD65 cells. Metabolomics samples for each hormone treatment using a positive 1.2–fold change cutoff from vehicle. Bar charts generated using enrichment over representation analysis feature and SMPDB metabolite data base on Metaboanalyst. Enrichment ratio is calculated by Hits / Expected, where hits = observed hits; expected = expected hits.

Supplementary Figure S4

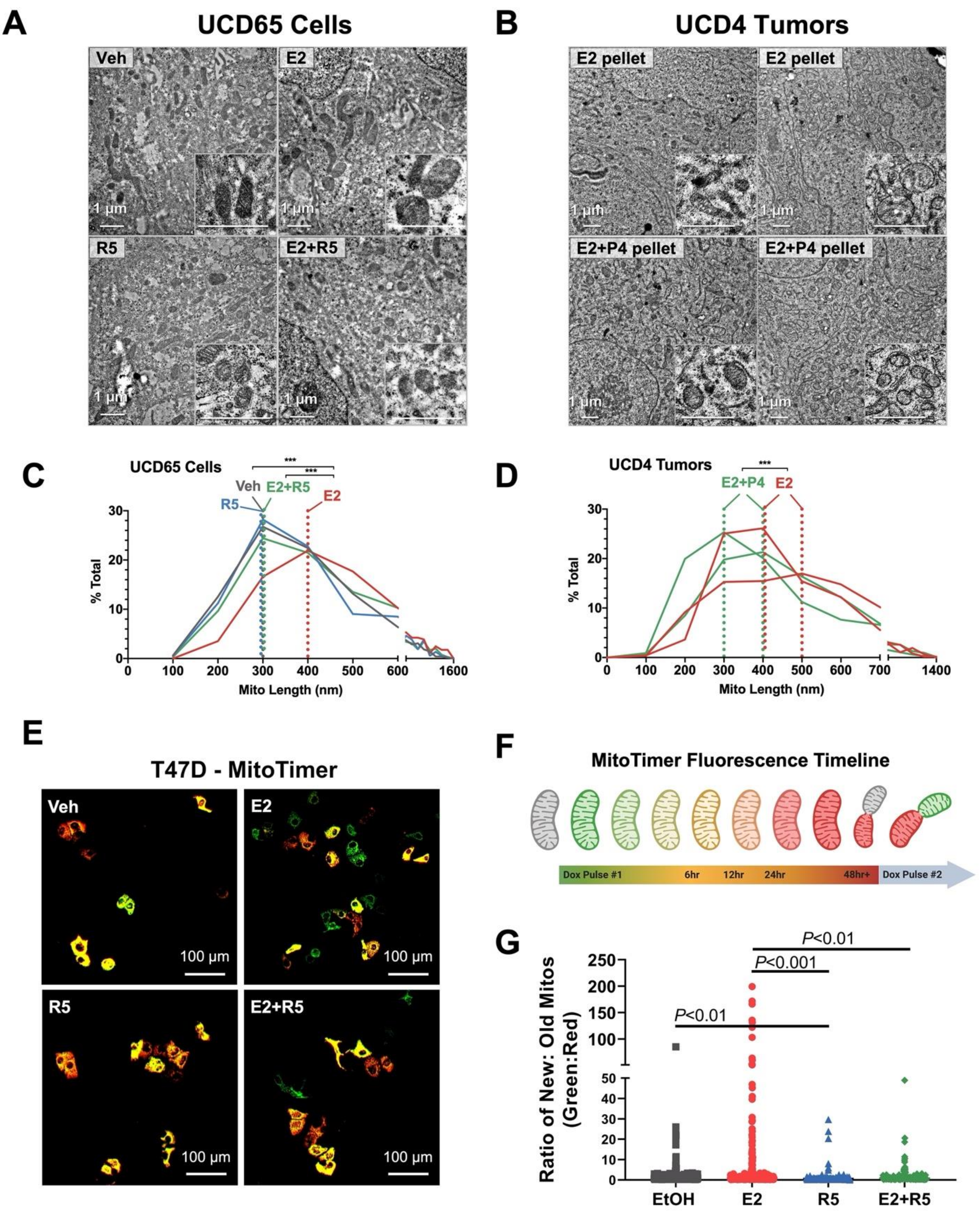

Figure S4. Steroid hormones alter mitochondrial morphology and length in UCD65 cells and UCD4 tumors. **A**) Cultured UCD65 cells treated with estradiol (E2, 10nM), promegesterone (R5, 10nM), combination (E2+R5, 10nM/10nM), or ethanol (vehicle) for 24 h were fixed, sectioned, and imaged using transmission electron microscopy. A representative image set is presented for each cell line and treatment (top). **B**) UCD4 xenograft tumors (2) from mice implanted hormone pellets containing E2 alone or E2+P4. A representative image set is presented for each tumor and treatment (top). **C**) Quantitation of mitochondrial length in UCD65 cells was measured along the longest axis in Fiji in >200 mitochondria across 10-14 fields per treatment. Histograms represent mitochondrial length corresponding to 100-nm bins. UCD65 cells, mode: Veh, E2+R5, R5 = 300 nm; E2 = 400 nm. **D**)

Quantitation of mitochondrial length in UCD4 tumors (individual tumors plotted separately) was measured and plotted as in C. UCD4 tumors, mode: E2+P4 tumor 1, 300; E2+P4 tumor 2, E2 tumor 1 = 400 nm; E2 tumor 2 = 500 nm. **P* <0.05, ***P* <0.01, ****P* <0.001 for the indicated comparisons using Kolmogorov-Smirnov test for frequency distributions comparing hormone treatments. **E**) MitoTimer: Representative fields from laser scanning confocal images taken at 10X magnification on T47D cells stably transduced with the doxycycline-inducible MitoTimer construct. Cells were pulsed with doxycycline for 1 h washed, treated with hormone for 48 h pulsed with Dox again, washout, and a final 24 h hormone treatment. Cells were fixed in 4% paraformaldehyde and imaged on an Olympus BX40 microscope at 10X magnification. **F**) Schematic of MitoTimer fluorescence over time with Dox pulses. **G**) Ratios of green to red fluorescent signal are shown and represent mean ± SEM of across 10 fields per treatment. Number of cells analyzed were vehicle (243), E2 (305), R5 (240), and E2+R5 (239). Data were log transformed to control for unequal variance, then analyzed by one-way ANOVA.

Supplementary Figure S5

A

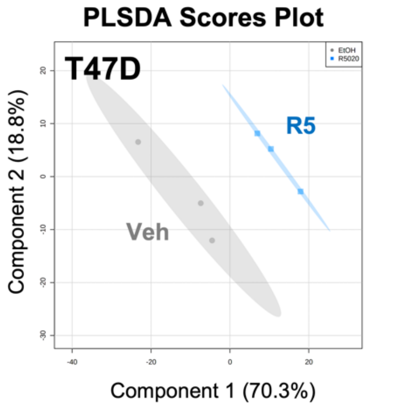

B

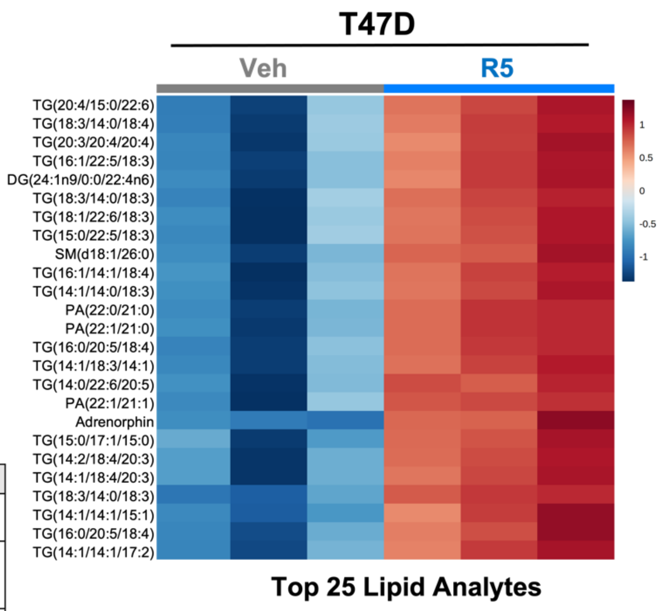

C

Pathway Analysis

Annotation	Category	Set	p-value	FDR
Glycerolipid metabolism (hsa00564)	KEGG pathways	3/6	3.85×10^{-05}	6.93×10^{-04}
Phospholipid Biosynthesis (SMP00025)	SMPDB Pathways (HMDB)	3/5	4.76×10^{-05}	2.38×10^{-04}
Metabolic pathways (hsa01100)	KEGG pathways	6/6	4.43×10^{-03}	1.29×10^{-02}

Figure S5. Progestin treatment increases total glycerolipids compared to vehicle in T47D cells. A) PLSDA scores plots of T47D lipidomic samples stratified by vehicle or promegesterone (R5, 10nM) treatment 24 h. Shaded areas indicate 95% confidence regions. **B**) Hierarchical clustering heatmaps of top 25 significant lipid analytes in T47D samples. Heatmaps were generated using Euclidean distance measure and Ward clustering method. Lipidomics data was normalized to protein per sample, log transformed, and auto-scaled in Metaboanalyst. **C**) Enrichment analysis of categorical annotations using MBROLE 2.0.

Supplementary Table S1 - Metabolite Enrichment Tables

Enrichment Table: T47D – E2 vs Veh

Metabolite Set	total	expected	hits	Raw p	Holm p	FDR
Pyrimidine Metabolism	59	3.17	12	2.80E-05	0.00274	0.00274
Purine Metabolism	74	3.97	13	6.46E-05	0.00627	0.00317
Pyruvate Metabolism	48	2.58	10	0.000118	0.0113	0.00348
Glutamate Metabolism	49	2.63	10	0.000142	0.0135	0.00348
Nucleotide Sugars Metabolism	20	1.07	6	0.000395	0.0371	0.00534
Lactose Synthesis	20	1.07	6	0.000395	0.0371	0.00534
Nicotinate and Nicotinamide Metabolism	37	1.99	8	0.000474	0.0436	0.00534
Glutathione Metabolism	21	1.13	6	0.00053	0.0482	0.00534
Pentose Phosphate Pathway	29	1.56	7	0.000545	0.049	0.00534
Urea Cycle	29	1.56	7	0.000545	0.049	0.00534
Warburg Effect	58	3.12	10	0.000617	0.0543	0.0055
Transfer of Acetyl Groups into Mitochondria	22	1.18	6	0.000699	0.0608	0.00571
Starch and Sucrose Metabolism	31	1.67	7	0.000844	0.0726	0.00636
Citric Acid Cycle	32	1.72	7	0.00104	0.088	0.00725
Arginine and Proline Metabolism	53	2.85	9	0.00135	0.113	0.0088
Glycerolipid Metabolism	25	1.34	6	0.00146	0.121	0.00896
Aspartate Metabolism	35	1.88	7	0.00182	0.149	0.0101
Cardiolipin Biosynthesis	11	0.591	4	0.00186	0.151	0.0101
Butyrate Metabolism	19	1.02	5	0.00243	0.194	0.0113
Mitochondrial Electron Transport Chain	19	1.02	5	0.00243	0.194	0.0113
Ethanol Degradation	19	1.02	5	0.00243	0.194	0.0113
Glycine and Serine Metabolism	59	3.17	9	0.00296	0.228	0.0127
Galactose Metabolism	38	2.04	7	0.00301	0.229	0.0127
Threonine and 2-Oxobutanoate Degradation	20	1.07	5	0.00311	0.233	0.0127
Tryptophan Metabolism	60	3.22	9	0.00334	0.247	0.0131

Enrichment Table: UCD4 – E2 vs Veh

Metabolite Set	total	expected	hits	Raw p	Holm p	FDR
Warburg Effect	58	2.15	12	3.02E-07	2.96E-05	2.96E-05
Urea Cycle	29	1.08	7	4.72E-05	0.00458	0.00231
Gluconeogenesis	35	1.3	7	0.000173	0.0166	0.00564
Glucose-Alanine Cycle	13	0.482	4	0.000909	0.0863	0.0223
Glycolysis	25	0.928	5	0.00166	0.156	0.0325
Glycine and Serine Metabolism	59	2.19	7	0.00454	0.422	0.063
Ammonia Recycling	32	1.19	5	0.0052	0.478	0.063
Citric Acid Cycle	32	1.19	5	0.0052	0.478	0.063
Carnitine Synthesis	22	0.816	4	0.0073	0.657	0.063
Transfer of Acetyl Groups into Mito	22	0.816	4	0.0073	0.657	0.063
Glutamate Metabolism	49	1.82	6	0.00752	0.662	0.063
Aspartate Metabolism	35	1.3	5	0.00772	0.672	0.063
Arginine and Proline Metabolism	53	1.97	6	0.0111	0.951	0.0833
Phenylalanine and Tyrosine Metabolism	28	1.04	4	0.0174	1	0.122
Pentose Phosphate Pathway	29	1.08	4	0.0197	1	0.128
Pyruvate Metabolism	48	1.78	5	0.0286	1	0.175
Glutathione Metabolism	21	0.779	3	0.0395	1	0.228
Malate-Aspartate Shuttle	10	0.371	2	0.05	1	0.272
Cardiolipin Biosynthesis	11	0.408	2	0.0597	1	0.308
Lysine Degradation	30	1.11	3	0.096	1	0.47
Tyrosine Metabolism	72	2.67	5	0.122	1	0.48
Beta-Alanine Metabolism	34	1.26	3	0.128	1	0.48
Alanine Metabolism	17	0.631	2	0.129	1	0.48
Purine Metabolism	74	2.75	5	0.133	1	0.48
Methylhistidine Metabolism	4	0.148	1	0.141	1	0.48

Enrichment Table: T47D – R5 vs Veh

Metabolite Set	total	expected	hits	Raw p	Holm p	FDR
Warburg Effect	58	2.72	11	3.29E-05	0.00322	0.00191
Pyrimidine Metabolism	59	2.77	11	3.90E-05	0.00378	0.00191
Purine Metabolism	74	3.47	12	7.09E-05	0.0068	0.00232
Glutathione Metabolism	21	0.984	6	0.000246	0.0234	0.00602
Starch and Sucrose Metabolism	31	1.45	7	0.000355	0.0334	0.00697
Gluconeogenesis	35	1.64	7	0.000787	0.0732	0.0128
Pyruvate Metabolism	48	2.25	8	0.00116	0.107	0.0137
Mitochondrial Electron Transport Chain	19	0.891	5	0.0013	0.118	0.0137
Glutamate Metabolism	49	2.3	8	0.00134	0.12	0.0137
Pentose Phosphate Pathway	29	1.36	6	0.00161	0.144	0.0137
Nucleotide Sugars Metabolism	20	0.938	5	0.00167	0.147	0.0137
Lactose Synthesis	20	0.938	5	0.00167	0.147	0.0137
Transfer of Acetyl Groups into Mito	22	1.03	5	0.00264	0.227	0.0193
Citric Acid Cycle	32	1.5	6	0.00276	0.235	0.0193
Aspartate Metabolism	35	1.64	6	0.00444	0.373	0.0276
Glycerolipid Metabolism	25	1.17	5	0.00479	0.398	0.0276
Glycolysis	25	1.17	5	0.00479	0.398	0.0276
Nicotinate and Nicotinamide Metabolism	37	1.73	6	0.00592	0.48	0.0322
Galactose Metabolism	38	1.78	6	0.00678	0.543	0.035
Threonine Degradation	20	0.938	4	0.0118	0.935	0.0545
Glycerol Phosphate Shuttle	11	0.516	3	0.0122	0.955	0.0545
Cardiolipin Biosynthesis	11	0.516	3	0.0122	0.955	0.0545
Pantothenate and CoA Biosynthesis	21	0.984	4	0.0141	1	0.0579
Fructose and Mannose Degradation	32	1.5	5	0.0142	1	0.0579
Phosphatidylethanolamine Biosynthesis	12	0.562	3	0.0158	1	0.0619

Enrichment Table: UCD4 – R5 vs Veh

Metabolite Set	total	expected	hits	Raw p	Holm p	FDR
Urea Cycle	29	0.34	3	0.00379	0.371	0.319
Aspartate Metabolism	35	0.41	3	0.00652	0.632	0.319
Lactose Synthesis	20	0.234	2	0.0213	1	0.695
Ammonia Recycling	32	0.375	2	0.0514	1	1
Phenylacetate Metabolism	9	0.105	1	0.101	1	1
Lactose Degradation	9	0.105	1	0.101	1	1
Glutamate Metabolism	49	0.574	2	0.109	1	1
Arginine and Proline Metabolism	53	0.621	2	0.125	1	1
Glucose-Alanine Cycle	13	0.152	1	0.143	1	1
Warburg Effect	58	0.68	2	0.145	1	1
Glycine and Serine Metabolism	59	0.691	2	0.149	1	1
Pyrimidine Metabolism	59	0.691	2	0.149	1	1
Spermidine and Spermine Biosynthesis	18	0.211	1	0.193	1	1
Catecholamine Biosynthesis	20	0.234	1	0.212	1	1
Purine Metabolism	74	0.867	2	0.213	1	1
Glutathione Metabolism	21	0.246	1	0.221	1	1
Transfer of Acetyl Groups into Mito	22	0.258	1	0.231	1	1
Glycolysis	25	0.293	1	0.258	1	1
Amino Sugar Metabolism	33	0.387	1	0.326	1	1
Gluconeogenesis	35	0.41	1	0.343	1	1
Nicotinate and Nicotinamide Metabolism	37	0.434	1	0.359	1	1
Galactose Metabolism	38	0.445	1	0.366	1	1
Sphingolipid Metabolism	40	0.469	1	0.382	1	1
Tyrosine Metabolism	72	0.844	1	0.585	1	1

Enrichment Table: T47D – E2+R5 vs Veh

Metabolite Set	total	expected	hits	Raw p	Holm p	FDR
Purine Metabolism	74	5.06	15	5.67E-05	0.00556	0.00556
Glutamate Metabolism	49	3.35	11	0.00025	0.0242	0.00711
Glutathione Metabolism	21	1.44	7	0.000279	0.0267	0.00711
Warburg Effect	58	3.96	12	0.00029	0.0276	0.00711
Pentose Phosphate Pathway	29	1.98	8	0.00043	0.0404	0.00843
Starch and Sucrose Metabolism	31	2.12	8	0.000707	0.0657	0.0115
Pyrimidine Metabolism	59	4.03	11	0.00137	0.126	0.0182
Nucleotide Sugars Metabolism	20	1.37	6	0.00149	0.135	0.0182
Gluconeogenesis	35	2.39	8	0.00169	0.152	0.0184
Nicotinate and Nicotinamide Metabolism	37	2.53	8	0.00247	0.22	0.0243
Pyruvate Metabolism	48	3.28	9	0.00375	0.33	0.0334
Glycerol Phosphate Shuttle	11	0.752	4	0.00459	0.399	0.0337
Cardiolipin Biosynthesis	11	0.752	4	0.00459	0.399	0.0337
Glycine and Serine Metabolism	59	4.03	10	0.00485	0.413	0.0337
Glycolysis	25	1.71	6	0.00516	0.433	0.0337
Methionine Metabolism	43	2.94	8	0.00665	0.552	0.0386
Mitochondrial Electron Transport Chain	19	1.3	5	0.00708	0.581	0.0386
Aspartate Metabolism	35	2.39	7	0.0074	0.6	0.0386
Arginine and Proline Metabolism	53	3.62	9	0.00749	0.6	0.0386
Lactose Synthesis	20	1.37	5	0.00895	0.707	0.0418
Glucose-Alanine Cycle	13	0.889	4	0.00895	0.707	0.0418
Urea Cycle	29	1.98	6	0.0111	0.856	0.0474
Pantothenate and CoA Biosynthesis	21	1.44	5	0.0111	0.856	0.0474
Galactose Metabolism	38	2.6	7	0.0118	0.883	0.0481
Transfer of Acetyl Groups into Mito	22	1.5	5	0.0136	1	0.0535

Enrichment Table: UCD4 – E2+R5 vs Veh

Metabolite Set	total	expected	hits	Raw p	Holm p	FDR
Warburg Effect	58	1.81	10	3.81E-06	0.000373	0.000373
Urea Cycle	29	0.906	5	0.0015	0.146	0.0737
Gluconeogenesis	35	1.09	5	0.0036	0.345	0.117
Glycolysis	25	0.781	4	0.00627	0.596	0.154
Amino Sugar Metabolism	33	1.03	4	0.017	1	0.308
Aspartate Metabolism	35	1.09	4	0.0208	1	0.308
Lactose Synthesis	20	0.625	3	0.022	1	0.308
Transfer of Acetyl Groups into Mito	22	0.688	3	0.0285	1	0.349
Pentose Phosphate Pathway	29	0.906	3	0.0583	1	0.584
Glucose-Alanine Cycle	13	0.406	2	0.0596	1	0.584
Ammonia Recycling	32	1	3	0.0743	1	0.591
Citric Acid Cycle	32	1	3	0.0743	1	0.591
Arginine and Proline Metabolism	53	1.66	4	0.0784	1	0.591
Pyrimidine Metabolism	59	1.84	4	0.107	1	0.729
Galactose Metabolism	38	1.19	3	0.112	1	0.729
Methylhistidine Metabolism	4	0.125	1	0.119	1	0.731
Nucleotide Sugars Metabolism	20	0.625	2	0.127	1	0.731
Glutathione Metabolism	21	0.656	2	0.137	1	0.749
Carnitine Synthesis	22	0.688	2	0.148	1	0.765
Pyruvate Metabolism	48	1.5	3	0.186	1	0.91
Biotin Metabolism	8	0.25	1	0.225	1	1
Phenylacetate Metabolism	9	0.281	1	0.249	1	1
Lactose Degradation	9	0.281	1	0.249	1	1
Starch and Sucrose Metabolism	31	0.969	2	0.252	1	1
Glycine and Serine Metabolism	59	1.84	3	0.279	1	1

Table S1. T47D and UCD4 Metabolite Enrichment Tables. Metabolite Enrichment Analysis for each hormone treatment using a positive 1.2−fold change cutoff from vehicle in T47D and UCD4 cells. Tables generated using enrichment over representation analysis feature and SMPDB metabolite data base on Metaboanalyst. Enrichment ratio is calculated by Hits / Expected, where hits = observed hits; expected = expected hits.

Supplementary Table S2 - ORA Tables

ORA Table: T47D – E2 vs Veh

ID	GeneRatio	BgRatio	pvalue	p.adjust	qvalue	Count
HALLMARK_MYC_TARGETS_V1	99/758	181/3314	1.09E-21	5.47E-20	4.95E-20	99
HALLMARK_MYC_TARGETS_V2	42/758	49/3314	8.03E-21	2.01E-19	1.82E-19	42
HALLMARK_ESTROGEN_RESPONSE_EARLY	98/758	187/3314	1.15E-19	1.91E-18	1.73E-18	98
HALLMARK_ESTROGEN_RESPONSE_LATE	77/758	175/3314	1.54E-10	1.93E-09	1.75E-09	77
HALLMARK_UNFOLDED_PROTEIN_RESPONSE	34/758	102/3314	9.28E-03	8.52E-02	7.71E-02	34
HALLMARK_E2F_TARGETS	56/758	185/3314	1.02E-02	8.52E-02	7.71E-02	56
HALLMARK_MTORC1_SIGNALING	53/758	181/3314	2.38E-02	0.1699	0.1538	53
HALLMARK_UV_RESPONSE_UP	38/758	132/3314	6.37E-02	0.3981	0.3604	38
HALLMARK_WNT_BETA_CATENIN_SIGNALING	12/758	37/3314	0.1180	0.6175	0.5590	12
HALLMARK_G2M_CHECKPOINT	50/758	188/3314	0.1235	0.6175	0.5590	50
HALLMARK_TNFA_SIGNALING_VIA_NFKB	41/758	154/3314	0.1501	0.6395	0.5789	41
HALLMARK_IL2_STAT5_SIGNALING	37/758	138/3314	0.1535	0.6395	0.5789	37
HALLMARK_ADIPOGENESIS	43/758	164/3314	0.1703	0.6549	0.5929	43
HALLMARK_NOTCH_SIGNALING	8/758	28/3314	0.2999	1.0000	0.9053	8
HALLMARK_P53_PATHWAY	18/758	92/3314	0.813	1.000	0.905	18

ORA Table: UCD4 – E2 vs Veh

ID	GeneRatio	BgRatio	pvalue	p.adjust	qvalue	Count
HALLMARK_E2F_TARGETS	100/496	185/3314	6.91E-38	3.46E-36	2.98E-36	100
HALLMARK_G2M_CHECKPOINT	85/496	188/3314	8.19E-25	2.05E-23	1.77E-23	85
HALLMARK_MYC_TARGETS_V1	76/496	181/3314	9.12E-20	1.52E-18	1.31E-18	76
HALLMARK_ESTROGEN_RESPONSE_EARLY	72/496	187/3314	3.04E-16	3.79E-15	3.28E-15	72
HALLMARK_ESTROGEN_RESPONSE_LATE	56/496	175/3314	3.90E-09	3.90E-08	3.36E-08	56
HALLMARK_MTORC1_SIGNALING	51/496	181/3314	1.86E-06	1.55E-05	1.34E-05	51
HALLMARK_MYC_TARGETS_V2	16/496	49/3314	1.38E-03	9.84E-03	8.49E-03	16
HALLMARK_MITOTIC_SPINDLE	40/496	194/3314	1.77E-02	0.1108	9.56E-02	40
HALLMARK_UNFOLDED_PROTEIN_RESPONSE	23/496	102/3314	2.50E-02	0.1386	0.1196	23
HALLMARK_UV_RESPONSE_UP	26/496	132/3314	7.98E-02	0.3867	0.3338	26
HALLMARK_EPITHELIAL_MESENCHYMAL_TRANSITION	25/496	127/3314	8.51E-02	0.3867	0.3338	25
HALLMARK_REACTIVE_OXYGEN_SPECIES_PATHWAY	8/496	39/3314	0.2194	0.8966	0.7739	8
HALLMARK_HEDGEHOG_SIGNALING	6/496	28/3314	0.2331	0.8966	0.7739	6
HALLMARK_ANGIOGENESIS	4/496	19/3314	0.3141	1.0000	0.8632	4
HALLMARK_UV_RESPONSE_DN	15/496	121/3314	0.8250	1	0.8632	15

ORA Table: T47D – R5 vs Veh

ID	GeneRatio	BgRatio	pvalue	p.adjust	qvalue	Count
HALLMARK_TNFA_SIGNALING_VIA_NFKB	20/144	154/3314	6.86E-06	3.23E-04	2.67E-04	20
HALLMARK_FATTY_ACID_METABOLISM	13/144	125/3314	2.61E-03	6.13E-02	5.08E-02	13
HALLMARK_ANDROGEN_RESPONSE	10/144	88/3314	4.31E-03	6.75E-02	5.59E-02	10
HALLMARK_EPITHELIAL_MESENCHYMAL_TRANSITION	12/144	127/3314	8.24E-03	9.06E-02	7.51E-02	12
HALLMARK_HYPOXIA	14/144	162/3314	9.64E-03	9.06E-02	7.51E-02	14
HALLMARK_COAGULATION	8/144	74/3314	1.39E-02	9.39E-02	7.78E-02	8
HALLMARK_APOPTOSIS	11/144	120/3314	1.40E-02	9.39E-02	7.78E-02	11
HALLMARK_MYOGENESIS	11/144	134/3314	2.95E-02	0.1590	0.1318	11
HALLMARK_ESTROGEN_RESPONSE_EARLY	14/144	187/3314	3.04E-02	0.1590	0.1318	14
HALLMARK_ESTROGEN_RESPONSE_LATE	13/144	175/3314	3.85E-02	0.1808	0.1498	13
HALLMARK_IL6_JAK_STAT3_SIGNALING	5/144	49/3314	5.94E-02	0.2538	0.2104	5
HALLMARK_XENOBIOTIC_METABOLISM	10/144	141/3314	8.37E-02	0.3140	0.2602	10
HALLMARK_REACTIVE_OXYGEN_SPECIES_PATHWAY	4/144	39/3314	8.68E-02	0.3140	0.2602	4
HALLMARK_UV_RESPONSE_UP	9/144	132/3314	0.1176	0.3947	0.3271	9
HALLMARK_IL2_STAT5_SIGNALING	7/144	138/3314	0.3929	0.7386	0.6121	7

ORA Table: UCD4 – R5 vs Veh

ID	GeneRatio	BgRatio	pvalue	p.adjust	qvalue	Count
HALLMARK_TNFA_SIGNALING_VIA_NFKB	24/143	154/3314	1.77E-08	7.98E-07	6.91E-07	24
HALLMARK_IL2_STAT5_SIGNALING	14/143	138/3314	2.12E-03	3.61E-02	3.12E-02	14
HALLMARK_ESTROGEN_RESPONSE_EARLY	17/143	187/3314	2.41E-03	3.61E-02	3.12E-02	17
HALLMARK_XENOBIOTIC_METABOLISM	13/143	141/3314	6.97E-03	7.85E-02	6.79E-02	13
HALLMARK_HYPOXIA	14/143	162/3314	9.08E-03	8.17E-02	7.07E-02	14
HALLMARK_ESTROGEN_RESPONSE_LATE	14/143	175/3314	1.72E-02	0.1292	0.1118	14
HALLMARK_P53_PATHWAY	13/143	175/3314	3.66E-02	0.2356	0.2039	13
HALLMARK_CHOLESTEROL_HOMEOSTASIS	6/143	62/3314	4.91E-02	0.2760	0.2389	6
HALLMARK_MYOGENESIS	10/143	134/3314	6.14E-02	0.3071	0.2658	10
HALLMARK_UV_RESPONSE_DN	9/143	121/3314	7.52E-02	0.3292	0.2849	9
HALLMARK_ANDROGEN_RESPONSE	7/143	88/3314	8.33E-02	0.3292	0.2849	7
HALLMARK_KRAS_SIGNALING_DN	8/143	108/3314	9.22E-02	0.3292	0.2849	8
HALLMARK_EPITHELIAL_MESENCHYMAL_TRANSITION	9/143	127/3314	0.0951	0.3292	0.2849	9
HALLMARK_FATTY_ACID_METABOLISM	8/143	125/3314	0.1694	0.5422	0.4693	8
HALLMARK_KRAS_SIGNALING_UP	5/143	115/3314	0.5596	0.8831	0.7643	5

ORA Table: T47D – E2+R5 vs Veh

ID	GeneRatio	BgRatio	pvalue	p.adjust	qvalue	Count
HALLMARK_MYC_TARGETS_V1	112/938	181/3314	3.29E-22	1.64E-20	1.35E-20	112
HALLMARK_MYC_TARGETS_V2	41/938	49/3314	6.62E-16	1.65E-14	1.36E-14	41
HALLMARK_ESTROGEN_RESPONSE_EARLY	95/938	187/3314	1.88E-11	3.13E-10	2.57E-10	95
HALLMARK_OXIDATIVE_PHOSPHORYLATION	78/938	165/3314	8.63E-08	1.08E-06	8.86E-07	78
HALLMARK_ADIPOGENESIS	73/938	164/3314	4.06E-06	3.40E-05	2.80E-05	73
HALLMARK_MTORC1_SIGNALING	79/938	181/3314	4.09E-06	3.40E-05	2.80E-05	79
HALLMARK_ESTROGEN_RESPONSE_LATE	72/938	175/3314	1.21E-04	8.62E-04	7.08E-04	72
HALLMARK_REACTIVE_OXYGEN_SPECIES_PATHWAY	22/938	39/3314	1.98E-04	1.24E-03	1.02E-03	22
HALLMARK_UNFOLDED_PROTEIN_RESPONSE	45/938	102/3314	3.85E-04	2.14E-03	1.75E-03	45
HALLMARK_FATTY_ACID_METABOLISM	48/938	125/3314	8.26E-03	4.13E-02	3.39E-02	48
HALLMARK_ANDROGEN_RESPONSE	33/938	88/3314	3.68E-02	0.1672	0.1372	33
HALLMARK_APOPTOSIS	40/938	120/3314	0.1274	0.5306	0.4357	40
HALLMARK_NOTCH_SIGNALING	11/938	28/3314	0.1397	0.5373	0.4411	11
HALLMARK_TNFA_SIGNALING_VIA_NFKB	49/938	154/3314	0.1835	0.6554	0.5381	49
HALLMARK_ALLOGRAFT_REJECTION	18/758	92/3314	0.8126	1.0000	0.9053	18

ORA Table: UCD4 – E2+R5 vs Veh

ID	GeneRatio	BgRatio	pvalue	p.adjust	qvalue	Count
HALLMARK_ESTROGEN_RESPONSE_EARLY	59/283	187/3314	6.49E-21	3.24E-19	2.80E-19	59
HALLMARK_HYPOXIA	37/283	162/3314	9.45E-09	2.36E-07	2.04E-07	37
HALLMARK_ESTROGEN_RESPONSE_LATE	37/283	175/3314	8.50E-08	1.23E-06	1.06E-06	37
HALLMARK_TNFA_SIGNALING_VIA_NFKB	34/283	154/3314	9.85E-08	1.23E-06	1.06E-06	34
HALLMARK_GLYCOLYSIS	27/283	169/3314	8.58E-04	8.58E-03	7.41E-03	27
HALLMARK_IL2_STAT5_SIGNALING	21/283	138/3314	5.81E-03	4.84E-02	4.18E-02	21
HALLMARK_MYOGENESIS	20/283	134/3314	8.74E-03	6.24E-02	5.39E-02	20
HALLMARK_EPITHELIAL_MESENCHYMAL_TRANSITION	18/283	127/3314	2.08E-02	0.1297	0.1120	18
HALLMARK_UV_RESPONSE_DN	17/283	121/3314	2.62E-02	0.1457	0.1257	17
HALLMARK_P53_PATHWAY	20/283	175/3314	0.1057	0.5284	0.4561	20
HALLMARK_APICAL_JUNCTION	18/283	157/3314	0.1179	0.5358	0.4625	18
HALLMARK_UV_RESPONSE_UP	15/283	132/3314	0.1523	0.5956	0.5141	15
HALLMARK_CHOLESTEROL_HOMEOSTASIS	8/283	62/3314	0.1549	0.5956	0.5141	8
HALLMARK_NOTCH_SIGNALING	4/283	28/3314	0.2128	0.6997	0.6040	4
HALLMARK_HEDGEHOG_SIGNALING	3/283	28/3314	0.4328	0.8642	0.7459	3

Table S2. T47D and UCD4 RNA-seq ORA Tables. Over-representation upregulated pathway analysis (ORA) tables on T47D and UCD4 cells for each hormone treatment compared against vehicle. Tables generated in CU Anschutz Genomics Core Shiny App using the molecular signatures data base (MSigDB) hallmark gene set collection.
